# Responding to the challenge of untreatable gonorrhea: ETX0914, a first-in-class agent with a distinct mechanism-of-action against bacterial Type II topoisomerases

**DOI:** 10.1038/srep11827

**Published:** 2015-07-14

**Authors:** Gregory S. Basarab, Gunther H. Kern, John McNulty, John P. Mueller, Kenneth Lawrence, Karthick Vishwanathan, Richard A. Alm, Kevin Barvian, Peter Doig, Vincent Galullo, Humphrey Gardner, Madhusudhan Gowravaram, Michael Huband, Amy Kimzey, Marshall Morningstar, Amy Kutschke, Sushmita D. Lahiri, Manos Perros, Renu Singh, Virna J. A. Schuck, Ruben Tommasi, Grant Walkup, Joseph V. Newman

**Affiliations:** 1 Department of Chemistry, Drug Discovery and Development Center, University of Cape Town, Rondebosch 7701, South Africa; 2 AstraZeneca R&D Boston, Infection iMed, 35 Gatehouse Dr. Waltham, MA 02415 USA; 3 Shire Pharmaceuticals, 300 Shire Way, Lexington, MA 02421; 4 Entasis Therapeutics, 35 Gatehouse Drive Suite E0, Waltham, MA 02415 USA; 5 Albany Molecular Research Inc., 26 Corporate Circle, Albany, NY 12203; 6 Center for Drug Evaluation and Research, U.S. FDA, 10903 New Hampshire Avenue, Silver Spring, MD 20993; 7 JMI Laboratories, 345 Beaver Kreek Centre, Suite A, North Liberty, IA 52317; 8 Broad Institute, 415 Main St., Cambridge, MA 02142; 9 Norvartis Pharmaceutical Corporation, Bldg. 335, Office 3104B, One Health Plaza, East Hanover, NJ 07936-1080.

## Abstract

With the diminishing effectiveness of current antibacterial therapies, it is critically important to discover agents that operate by a mechanism that circumvents existing resistance. ETX0914, the first of a new class of antibacterial agent targeted for the treatment of gonorrhea, operates by a novel mode-of-inhibition against bacterial type II topoisomerases. Incorporating an oxazolidinone on the scaffold mitigated toxicological issues often seen with topoisomerase inhibitors. Organisms resistant to other topoisomerase inhibitors were not cross-resistant with ETX0914 nor were spontaneous resistant mutants to ETX0914 cross-resistant with other topoisomerase inhibitor classes, including the widely used fluoroquinolone class. Preclinical evaluation of ETX0914 pharmacokinetics and pharmacodynamics showed distribution into vascular tissues and efficacy in a murine *Staphylococcus aureus* infection model that served as a surrogate for predicting efficacious exposures for the treatment of *Neisseria gonorrhoeae* infections. A wide safety margin to the efficacious exposure in toxicological evaluations supported progression to Phase 1. Dosing ETX0914 in human volunteers showed sufficient exposure and minimal adverse effects to expect a highly efficacious anti-gonorrhea therapy.

There have been repeated calls over the last few decades for the discovery and development of novel antibacterial agents to address widespread resistance to current treatment regimens^1^,^2^. Since the mid-1960’s, only three agents introduced to the clinic have new modes-of-action or modes-of-inhibition, namely daptomycin, linezolid and bedaquiline, the first two for the treatment of resistant Gram-positive bacterial infections and the latter for the treatment of tuberculosis^3^. Spiropyrimidinetriones are a new class of bacterial type II topoisomerase inhibitors with a novel mode-of-inhibition that avoids cross-resistance to antibacterial agents currently in clinical use and offers the promise of additional oral treatment options for patients and prescribers^4^,^5^,^6^. ETX0914 (**1,** Fig. 1), a candidate drug in this class, displays an antibacterial spectrum that includes Gram-positive pathogens such as *Staphylococcus* spp. and *Streptococcus* spp. and fastidious Gram-negative pathogens such as *Haemophilus influenzae*, *Moraxella catarrhalis* and *Neisseria gonorrhoeae*^7^,^8^,^9^. With this profile, ETX0914 has the potential to treat acute and chronic bacterial skin and skin structure infections, acute respiratory tract infections and uncomplicated gonococcal infections including those resistant to other antibacterial classes (Table S1). Treatment of gonorrhea will be addressed initially with ETX0914, and establishing its clinical safety and efficacy will provide an opportunity to pursue other indications including extended oral treatment of patients with persistent infections caused by *S. aureus*. Such infections include bone, chronic osteomyelitis and infections caused by prosthetic joints, valves and implantable catheters^10^.

In 2013 the United States Centers for Disease Control and Prevention (CDC) released a report classifying drug-resistant *N. gonorrhoeae* as an “Urgent Threat” requiring aggressive action as treatment failures against uncomplicated gonorrhea accumulate^11^,^12^. Drugs that are no longer recommended as monotherapy for the treatment of gonorrhea due to resistance include sulfanilamides, penicillins, tetracyclines, and fluoroquinolones^2^. Recent studies have documented clinical failures caused by *N. gonorrhoeae* resistant to the current first-line treatment options for gonorrhea including azithromycin and the extended spectrum cephalosporins, cefixime and ceftriaxone, raising concerns that gonorrhea will become untreatable^13^,^14^,^15^. The availability of an effective and well-tolerated oral agent is thus desperately needed to reduce the dissemination of multidrug-resistant *N. gonorrhoeae*.

ETX0914 follows three other classes of antibacterial agents (Fig. 1) that target the type II topoisomerases, DNA gyrase and topoisomerase IV (Topo IV). Both topoisomerases are heterodimeric A_2_B_2_ enzymes consisting of two units (A = GyrA or ParC) responsible for cleavage/re-ligation of the leading DNA strand, and two units (B = GyrB or ParE) responsible for ATP hydrolysis needed for passing the lagging DNA strand through the cleaved leading DNA strand. Fluoroquinolones, including ciprofloxacin **2**, have been the most widely used class of antibacterials inhibiting topoisomerases. However, decades of fluoroquinolone use has led to extensive target site mediated resistance limiting the utility against *S. aureus* and *N. gonorrhoeae* among other bacteria^16^,^17^. A second class of topoisomerase inhibitors known as NBTIs (novel bacterial topoisomerase inhibitors) have not, as of yet, been approved for therapeutic use, but there are three compounds (including compound **3** of Fig. 1) that have entered Phase 1 clinical trials and have the potential to circumvent cross-resistance^18^,^19^,^20^. The third class of inhibitors compete with ATP in the GyrB/ParE subunits and represent a third mode-of-inhibition relative to fluoroquinolones and NBTIs. Novobiocin **4** is the only member of this class that has been commercialized despite a diverse array of GyrB/ParE ATP competitive chemotypes that have been investigated^21^,^22^. However, novobiocin never attained an extensive market presence, and its manufacture was discontinued in 1997 as its utility was superseded by other antibacterial agents of greater safety and effectiveness^23^.

Therefore, in addition to a low potential for cross-resistance to current regimens, an agent advancing to clinical trials should demonstrate a sufficient margin between adverse effects in animal models and predicted efficacious doses. ETX0914 shows key attributes to support its development as a new antibacterial treatment option with a novel mode-of-inhibition relative to fluoroquinolones, NBTIs and novobiocin. The extensive preclinical and early clinical evaluations performed to support its progression to Phase 2 clinical trials are discussed in this manuscript.

## Results and Discussion

### The benzisoxazole 3-position of the spiropyrimidinetrione scaffold is amenable for SAR exploration

Spiropyrimidinetriones were assembled via a key tertiary amino effect reaction affording a spirocyclic architecture (Figure S1). Previously, the evolution to the promising lead compound **5** (Fig. 2) was described including its activity against quinolone susceptible and quinolone resistant *S. aureus*, *S. pneumoniae* and *H. influenzae* among other pathogens^5^. The solubility and fraction unbound (f_u_) to plasma protein binding (PPB) of **5** were sufficiently high to warrant an extensive analog program. Pharmacokinetic (PK) properties of **5** were also favorable as seen by the low plasma clearance (CL_p_) and good bioavailability in dog. The subsequent analog program around **5** established a rather restricted structural latitude for antibacterial activity. The spirocyclic pyrimidinetrione of the scaffold could not be altered by substitution, isosteric replacement or truncation. The specific (2*R*, 4a*S*, 4a*S*)-stereochemistry around the fused morpholine ring was required for activity. The morpholine ring oxygen can be replaced with carbon, but at the expense of solubility, and the two methyl substituents proved to be optimal. The benzisoxazole fluorine atom was also optimal; though replacement with a chlorine atom afforded similar activity, it increased lipophilicity and decreased solubility and f_u_^5^. However, the diversity of substituents tolerated at the benzisoxazole 3-position was quite high^24^,^25^ and is exemplified by the five contrasting R-groups, all giving low MIC values across pathogens of interest (Fig. 2).

The broad tolerance for substitution at the benzisoxazole 3-position for antibacterial activity enabled diversification and optimization toward compounds that might mitigate undesirable mammalian toxicological effects. The *in vitro* toxicological data associated with compounds **6–8** and ETX0914 show a general progression to compounds of greater safety (Fig. 2). Genotoxicity, as manifested *in vitro* through a mouse micronucleus aberration assay (MMA) and a mouse lymphoma assay as well as *in vivo* through a rat MMA, has been of concern for some fluoroquinolones including gemifloxacin^26^. Compound **5** showed a moderate, but measurable genotoxicity signal at 100 μM in the *in vitro* MMA; for comparison, the values for gemifloxacin and ciprofloxacin were 6 and 100 μM, respectively. Compound **6** with an *N*-linked triazole ring showed higher antibacterial activity than **1**, but it induced micronucleation similar to gemifloxacin. Furthermore, dosing **6** at 100 mg/kg/day for 14 days in the rat led to haematologic effects and a potential for a reduction in bone marrow function as manifested by decreases in reticulocytes, neutrophils, lymphocytes and monocytes (Figure S2). Hence, subsequent analogs were assessed for potential bone marrow suppression by measuring the growth inhibition of mammalian erythroid and myeloid cell lines *in vitro* (Fig. 2). Bone marrow suppression leading to anemia, leukopenia or thrombocytopenia has been seen with some fluoroquinolones including gemifloxacin and with linezolid, hence their inclusion as comparators for erythroid and myeloid IC_50_ determinations^27^,^28^. Generally, planar aromatic and carboxamide substitutions at the benzisoxazole 3-position proved more problematic than groups with the capability to introduce asymmetric centers through sp^3^ hybridization. Higher sp^3^ content of drug candidates has been correlated with clinical success, attributed, in part, to greater selectivity for desired targets and lower promiscuity toward off-targets^29^. *N*-linked oxazolidinones on the benzisoxazole 3-position in particular, as exemplified with ETX0914, stood out as having the best profile in toxicity related *in vitro* assays (Fig. 2 and Table S2). The lack of *in vitro* inhibition of erythroid and myeloid cell lines for ETX0914 at the highest concentration tested translated to a good haematology profile in rats (compare ETX0914 with **6** and linezolid, Figure S2). Furthermore, there were no significant changes in platelet count with ETX0914 in contrast to the thrombocytopenia seen for some fluoroquinolones and for linezolid (data not shown)^30^,^31^. Some fluoroquinolones induce QT prolongation leading to arrhythmia and torsades de pointes, which have been correlated with binding to the hERG K^+^ channel. Such QT prolongation has been cited for the withdrawal of at least two fluoroquinolones, sparfloxacin and grepafloxacin, from the market^32^,^33^,^34^. In contrast, ETX0914 also showed high IC_50_ values against a variety of ion channels associated with the action potential in cardiac cells including hERG, indicating a low likelihood for inducing arrhythmia (Table S2). Margins greater than 10-fold were achieved relative to the C_max_ at the highest 4000 mg dose administered in Phase 1 studies (see below). With such favorable *in vitro* toxicological properties and DMPK characteristics, ETX0914 was thereby selected for clinical development.

### ETX0914 exhibits a different mode-of-inhibition from fluoroquinolones

To position a spiropyrimidinetrione as an effective clinical agent, it needs to be demonstrated that a unique mode-of-inhibition against DNA gyrase and Topo IV would mitigate the potential for cross-resistance with other topoisomerase inhibitors. DNA gyrase and Topo IV are homologous enzymes that maintain DNA topology during replication by introducing negative supercoils (primarily by DNA gyrase) and by decatenating DNA (primarily by Topo IV). The complex course of the catalytic mechanism encompasses multiple conformational states and offers multiple possibilities for inhibition^35^,^36^. Previous work with *S. aureus* and *Escherichia coli* topoisomerases demonstrated that ciprofloxacin inhibited the re-ligation reaction of DNA gyrase by stabilizing a complex with DNA through a non-catalytic Mg^2+^ that interacts with an aspartate/glutamate (E88, Fig. 3) and a serine (S84) residue of GyrA through associated water molecules^37^. ETX0914 also stabilized the DNA cleaved complexes of DNA gyrase and Topo IV from *S. aureus* as manifested by the accumulation of linear DNA in gel-based assays (Figure S3). To differentiate between the ETX0914 and ciprofloxacin mode-of-inhibition, the effect of removing Mg^2+^ by addition of EDTA to inhibitor stabilized DNA-protein-cleaved complexes was investigated^38^,^39^. For ciprofloxacin stabilized complexes of DNA gyrase, re-ligation was observed at free Mg^2+^ concentrations between 0.1 μM and 2 mM as ascertained by an increase of intact circular DNA. In contrast, re-ligation remained blocked for the complex stabilized by ETX0914 at all Mg^2+^ concentrations (Figure S3a). A similar trend was seen for the cleaved complexes of Topo IV with ciprofloxacin and ETX0914 (Figure S3b). Hence, Mg^2+^ was not critically involved in the binding of ETX0914 to *S. aureus* topoisomerases differentiating its binding mode from that of ciprofloxacin. The differentiation of the role of Mg^2+^ offers a partial explanation for the susceptibility of fluoroquinolone resistant *S. aureus* to ETX0914 and would likely extend to the lack of cross-resistance for other bacterial species.

### Cross-resistance is not observed between ETX0914 and other topoisomerase inhibitors

Table 1 shows clinical isolates in the AstraZeneca Research Collection (ARC) of *S. aureus* (ARC2381), *S. pneumoniae* (ARC2480) and *N. gonorrhoeae* (ARC4672, ARC4680 and ARC4676) that are resistant to fluoroquinolones due to three resistance determinants, S84, S85 and E88 (*S. aureus* numbering), but are fully susceptible to ETX0914. This follows previous studies where ciprofloxacin resistant *S. aureus* and *S. pneumoniae*, manifested by mutations in GyrA and ParC, were fully susceptible to spiropyrimidinetriones^5^,^40^. As described, S84 and E88 are associated with the Mg^2+^ chelated by fluoroquinolones; S_85_P is thought to alter the conformational integrity of the α-helix that spans S84 and E88, thereby altering ciprofloxacin binding (Fig. 3b). The three resistant isolates were also fully susceptible to novobiocin and the aminopiperidine NBTI, with the one exception being reduced susceptibility of ARC4680 to the NBTI. This reflects the proximity and/or partial overlap of the fluoroquinolone and NBTI binding sites. *S. aureus* and *S. pneumoniae* GyrB\ParE mutants (ARC3445 and ARC2800, respectively) with reduced novobiocin susceptibility due to changes in the ATP binding region were generated by incubating the bacteria with ATPase inhibitors on agar plates. These mutants were fully susceptible to ETX0914, consistent with the compound’s lack of inhibition of ATPase activity as measured in Malachite Green based assays of DNA gyrase or Topo IV^41^. Finally, a spontaneous NBTI resistant *S. aureus* mutant ARC2796 was derived from ARC516 and was not cross-resistant to ETX0914, nor was it cross-resistant to ciprofloxacin or novobiocin (Table 1). Therefore, the ETX0914 mode-of-inhibition for DNA gyrase is distinct from ciprofloxacin, novobiocin and the NBTI.

### Spontaneous mutants to ETX0914 are not cross-resistant

**Figure 1:**
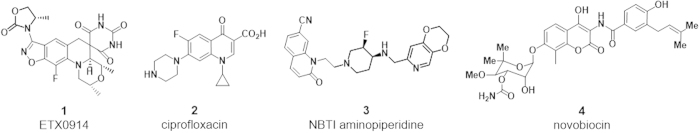
Structures of previously disclosed antibacterial inhibitors of DNA gyrase/Topo IV. The four compounds are representatives of four drug classes that operate by orthogonal modes-of-inhibition. ETX0914 is spiropyrimidinetrione with noteworthy activity versus Staphylococci and Streptococci and fastidious Gram-negative pathogens including *N. gonorrhoeae*. Ciprofloxacin **2** is one of dozens of fluoroquinolones that have been introduced into the clinic. NBTI **3** is a representative from an aminopiperidine class of agents with three reported compounds having progressed to human clinical trials, only one of which is known have progressed to patient trials. Novobiocin **4** is the only commercialized example of a broad class of DNA gyrase/Topo IV ATP competitive inhibitors.

**Figure 2:**
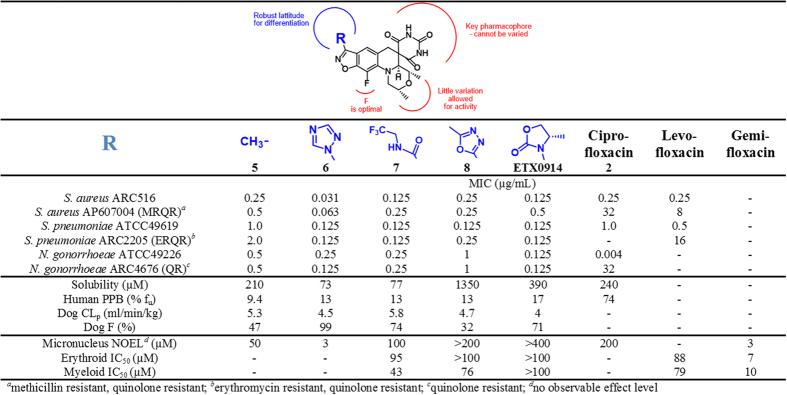
MIC, PK and key *in vitro* safety data are shown for five spiropyrimidinetriones variably substituted on the benzisoxazole 3-position. Structure-activity correlations show that the pyrimidinetrione and morpholine moieties cannot be varied without diminishing antibacterial potency or compromising co-dependent physical properties and PPB. Importantly, ETX0914 did not show a signal for genotoxicity or bone marrow toxicity at the highest concentrations tested. Three fluoroquinolones are included as comparators.

**Figure 3:**
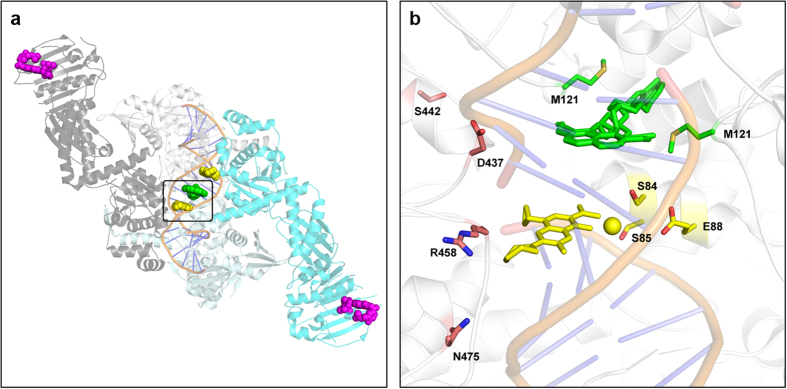
Orientation of topoisomerase resistance determinants and mutable sites to inhibitors of bacterial type II topoisomerases. (**a**) ‘Overhead’ view of composite DNA gyrase/Topo IV structure showing binding sites for ciprofloxacin **2** (2 molecules in yellow), NBTI **3** (green) and novobiocin **4** (2 molecules in magenta). The structural information was generated by overlaying pdb files of *S. aureus* GyrB bound to novobiocin (4URO), a three-gate structure of *S. pneumoniae* TopoIV (4I3H), a *S. aureus* structure with NBTI (2XCS) and a *S. aureus* structure with ciprofloxacin (2XCT). The region of the binding pocket used for expansion in (**b**) has been boxed. (**b**) Expansion of the DNA cleavage domain (2A) showing: ciprofloxacin (yellow sticks), the associated Mg^2+^ (yellow sphere), and resistance determinants GyrA: S84, S85 and E88 (yellow sticks); NBTI **3** (two orientations of the molecule, green sticks) and resistance determinant GyrA: M121 (green sticks); ETX0914 mutable sites: S442, D437, R458 and N475 (orange sticks). Resistance determinants and mutable sites are numbered by the *S. aureus* DNA gyrase sequence. The novobiocin T172 mutable sites are not shown.

To further investigate the mode-of-inhibition among the topoisomerase inhibitors, spontaneous resistant mutants to ETX0914 were assessed for cross-susceptibility in a *S. aureus* strain and two *N. gonorrhoeae* strains, one of which is fluoroquinolone resistant. The frequencies of resistance to ETX0914 were low for various strains of *S. aureus* (<1.1 × 10^−9^ at 4X MIC) and *N. gonorrhoeae* (1.5 × 10^−8^ to <5.2 × 10^−9^ at 4X MIC) relative to those seen for fluoroquinolones^7^,^9^,^42^,^43^. Sequence analysis of the resistant colonies revealed four mutable sites all in GyrB of DNA gyrase including the two sequence aligned D_437_N and D_429_N mutations^7^,^9^,^43^ from the two pathogens (Table 1). The mutants were fully susceptible to ciprofloxacin, novobiocin and the NBTI providing further evidence that spiropyrimidinetriones inhibit DNA gyrase as the mode-of-action and that the mode-of-inhibition differs from that of fluoroquinolones and other bacterial topoisomerase inhibitors. Collectively, the data showed that the four classes of compounds of Fig. 1 represent four orthogonal modes-of-inhibition wherein target-based resistance to any single one would not show the same levels of cross-resistance to the others. Figure. 3 maps the clinical resistance determinants and mutable sites leading to resistance for ETX0914, ciprofloxacin and the NBTI, all clustering around the interface of the topoisomerase DNA cleavage domain. Resistant mutations to ETX0914 have only been seen on GyrB: they are near the ciprofloxacin binding pocket but are located away from the ciprofloxacin resistance determinants and NBTI mutable sites.

### Pharmacokinetics and distribution predict high drug levels in relevant vascularised tissues

In hepatocytes, the intrinsic clearance (Cl_int_) of ETX0914 indicated that metabolism mediated clearance was low in human (1.0 μL/min/10^6^ cells) relative to dog, rat and mouse (Table S3). For both rat and dog, low Cl_int_ in hepatocytes was predictive of the observed *in vivo* plasma clearance (CL_p_) values of 22 and 4 mL/min/kg observed, whereas the mouse Cl_int_ predicted a significantly lower *in vivo* clearance than that observed (CL_p_ = 70 mL/min/kg). Pre-dosing with 1-aminobenzotriazole (ABT, a cytochrome P450 inactivator)^44^ lowered the CL_p_ nearly 5-fold to 15 mL/min/kg indicating that P450 metabolism contributed significantly to the high mouse CL_p_. The reason for the low turnover in mouse hepatocytes relative to the *in vivo* CL_p_ could not be determined. These results led to murine efficacy experiments being carried out in the presence of ABT. The observed low apparent clearance in human from the healthy volunteer study (see below) confirmed that the higher clearance observed in mice was specific to mice. In addition, higher bioavailability (F) indicating low first pass effects was recorded in dog (71%) and monkey (58%) relative to mouse (46%) and rat (34%). The half-life after oral and intravenous administration in both dog and monkey was similar, indicating that absorption was not a rate limiting process in these species. PPB of ^14^C-ETX0914 was independent of concentration between expected (and ultimately realized) clinically relevant concentrations of 1–50 μM and was similar across species, ranging from 17.0% free (human) to 22.1% free (mouse). Free plasma levels were used for the assessment of pharmacodynamic (PD) and toxicological effects (below).

Following a single 15 minute intravenous infusion of ^14^C-ETX0914 into male pigmented rats, radioactivity was distributed throughout the body (Figure S4). Highly perfused tissues showed the highest concentrations generally exceeding blood levels (Table S4). By 4 hours, radioactivity was confined to the liver, where it remained detectable for 48 hours. Therefore, biliary excretion of the radioactivity appeared to predominate, consistent with metabolism as a main route of elimination. In addition, the results of this study indicated a very low CNS penetration and no melanin or covalent binding of the parent compound and/or metabolites to any tissue (Table S4). ETX0914 accounted for ~90% of the circulating radioactivity in plasma consistent with the low CL_p_ observed in rats; therefore, distribution into soft tissues was most likely unmodified ETX0914. Hence, ETX0914 has the appropriate metabolic and tissue distribution characteristics in preclinical species to support the proposed clinical indications.

### AUC/MIC is the PK/PD index best-associated with ETX0914 efficacy

**Figure 4:**
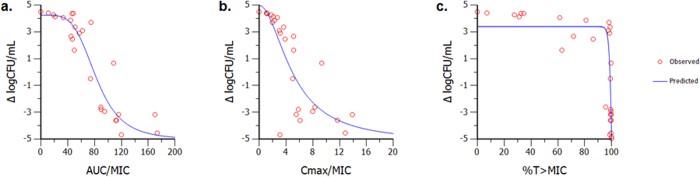
Relationship between AUC/MIC (**a**) C_max_/MIC (**b**) and %T > MIC (**c**) of ETX0914 against *S. aureus* ARC516 response in the hollow fiber infection model. R^2^ and weighted sum of the squared residual (WSSR) shown to indicate goodness of fit. Analysis of these data indicate that PK/PD index AUC/MIC is highly correlated to the efficacy of ETX0914.

The PK/PD index best-associated with ETX0914 efficacy was defined in a hollow fiber infection model (HFIM) against *S. aureus* ARC516, wherein non-protein bound plasma drug concentrations were simulated over 24 hours. Like other bacterial topoisomerase inhibitors^45^,^46^, the area under the curve (AUC) corrected to the pathogen MIC (AUC/MIC) was the PK/PD index most closely correlated for the response against *S. aureus*, with an R^2^ value of 0.95 as compared to C_max_/MIC (R^2^ = 0.88) and %T > MIC (R^2^ = 0.85) (Fig. 4).

### *S. aureus* infection models serve as a surrogate for *N. gonorrhoeae* infections

PK/PD targets have not been developed nor established for *N. gonorrhoeae* in animal studies due to spontaneous eradication of the bacterium; the existing animal model only provides a qualitative response negating a quantitative translation to clinical outcome^47^. To estimate a human efficacious exposure of ETX0914 for *N. gonorrhoeae*, a surrogate pathogen approach was undertaken utilizing PK/PD determinations from a mouse *S. aureus* neutropenic thigh model that has been shown to correlate with human clinical efficacy^48^. For ciprofloxacin and other fluoroquinolones, clinical efficacy correlated with a free AUC/MIC (fAUC/MIC) range of 20–40 for Gram-positive and Gram-negative pathogens in mouse thigh infection models^49^. By correcting for interspecies PK differences and the MIC of the pathogen tested, PK/PD magnitudes can be determined for preclinical species and extrapolated to clinical exposure requirements. Comparisons of the efficacious clinical doses of the fluoroquinolones ofloxacin and ciprofloxacin were utilized across the treatment regimens of both *S. aureus* and *N. gonorrhoeae* infections to assess the translatability of PK/PD indices (Table 2)^49^,^50^. Using the efficacious clinical dose of ciprofloxacin, exposure data from the product labels and the MIC_90_, the PK/PD index and magnitude observed to achieve efficacy in skin structure and gonococcal infections correlated well. Although the ofloxacin dose used to treat gonococcal infections resulted in a higher predicted PK/PD magnitude compared to the doses used to treat skin infections, there are reports of successful clinical treatment with ofloxacin when the MIC of the clinical isolate exceeded the MIC_90_^51^, indicating that the effective ofloxacin PK/PD magnitudes for the two organisms may be similar.

### ETX0914 was efficacious *in vivo* against clinical isolates of *S. aureus*

PK/PD magnitudes for ETX0914 were determined in the neutropenenic mouse thigh infection model^48^ using four *S. aureus* strains: MSSA ARC516, MRSA ATCC33591, USA100 NRS382 and USA300 NR538 (Table S5). USA100 and USA300 isolates have become epidemic in the hospital and community setting displaying a decreased susceptibility to an array of antimicrobial agents, including macrolides, fluoroquinolones, glycopeptides and tetracyclines. To the achieve exposure closer to that predicted in humans, mice were co-dosed with the hepatocyte metabolism inhibitor, ABT. Levofloxacin, ceftriaxone and linezolid were evaluated in the same animal model (Table S6). Linezolid lacks sufficient *in vitro* activity to expect clinical efficacy against *N. gonorrhoeae* and other Gram-negative organisms^52^, but it is the standard of care for the treatment of methicillin-resistant *S. aureus* and the observed PK/PD magnitude matched published work, corroborating the model^53^,^54^. Levofloxacin serves as a mechanistic comparator for topoisomerase inhibitors, and its PK/PD magnitude compared favorably with gatifloxacin, a fluoroquinolone previously tested against *S. aureus*^46^. The PK/PD magnitude for ceftriaxone, a current standard of care for *N. gonorrhoeae*, was taken from the literature^55^.

The translatability of the PK/PD targets from animal models to clinical utility was established by comparing the free plasma PK/PD magnitudes obtained for the reference compounds in the *S. aureus* neutropenic thigh model to the clinical PK/PD magnitudes associated with the efficacious clinical dose in complicated skin and skin structure infections^56^,^57^,^58^. For ceftriaxone, PK/PD targets, corrected for protein binding, were based on the prescribing information^59^ with final magnitudes calculated using the clinical two gram dose and the MIC_90_ of 8 μg/mL for *S. aureus*^60^. Overall, there was a good agreement between the preclinical and clinical PK/PD magnitudes associated with a stasis end point (Table S6) for the skin infections caused by *S. aureus*. Using the MIC values of ETX0914 against *S. aureus* and *N. gonorrhoeae* and the clinical doses suggests that efficacy can be translated across pathogens based on AUC/MIC targets. From this analysis, the efficacious human exposure for ETX0914 was estimated utilizing a PK/PD target that covers a mean fAUC/MIC of 66 (range 43–98, Table S5) for *S. aureus* in the mouse thigh model. This, combined with the MIC_90_ of ETX0914 for *N. gonorrhoeae* (0.12 μg/ml) and the human f_u_ of 17%, translated to a predicted efficacious mean AUC in humans of 49 μg*h/mL.

### Preclinical *in vivo* toxicology supports progression to Phase 1 trials

The favorable *in vitro* characteristics observed in ETX0914 related to drug toxicity were followed by extensive *in vivo* toxicology investigations, which is particularly important for a drug with a new chemotype and inhibitory mechanism. Data collected included clinical observations, body weight changes, food consumption, clinical pathology and histopathology on dosing rats and dogs. In the rat, ETX0914 was tolerated up to 1000 mg/kg/day (oral) for 28 days (Table S7). The rat NOAEL (no observable adverse effect level) was considered to be 200 mg/kg and correlated with a C_max_ of 107 μg/mL and an AUC_24h_ of 1070 μg*h/mL seen at day 27. The fAUC represents a 13-fold margin to the efficacious fAUC observed in the mouse thigh model. In the dog, doses of 500 mg/kg/day (oral gavage administration) were tolerated for 28 days. At this highest dose tested, tachycardia was observed, consistent with an IV telemetry dog study wherein decreases in blood pressure, contractility and PR interval and an increase in the heart rate were observed at 100 mg/kg. These effects were transient and fully reversible and, importantly, can be monitored in the clinical setting. There were no changes to other ECG intervals including QTc or QRS with ETX0914 administration. The dog NOAEL was established at 100 mg/kg PO for the 28 day study with a C_max_ of 85 μg/mL and AUC_24h_ of 870 μmol*h/mL measured on day 27. There were no significant changes in haematology or platelet count indicating anaemia or thrombocytopenia at the NOAEL in neither the rat nor dog,. Histopathology in both rat and dog at higher doses showed a variety of findings (Table S7). Overall, the preclinical data supported a successful IND submission to the FDA for single dose studies in healthy volunteers to support a single dose oral therapy for the treatment of uncomplicated gonorrhea.

### ETX0914 was well tolerated in human volunteers

A single center, multipart Phase 1 trial with an oral suspension of ETX0914 versus placebo was conducted in 48 healthy men and women of non-child bearing potential at doses ranging from 200–4000 mg. ETX0914 was absorbed relatively quickly under fasting conditions with a median T_max_ ranging from 1.5 to 2.26 hours in a dose independent fashion (Figure S5). Following C_max_, plasma drug concentrations declined in a mono-exponential manner. The terminal elimination phase started at approximately 4 hours, and over the 8 to 72 hour post-dose period, ETX0914 concentrations declined with a half life ranging from 5.2–6.25 hours. Less than 5% of the oral dose was excreted as parent drug in urine. Exposures escalated nearly proportionally to dose at the lower doses with a reduction in proportionality at the higher doses, suggesting a rate limitation due to absorption or solubility. When 3000 mg of ETX0914 was administered, more than a 40% increase in AUC (from 195 to 281 μg*h/mL) was seen if the drug was preceded by a high fat meal. The C_max_ increased from 22.9 to 24.0 μg/mL and the T_max_ from 2.2 to 4.0 hours with food in line with solubility/absorption being rate limiting at the higher dose.

**Figure 5:**
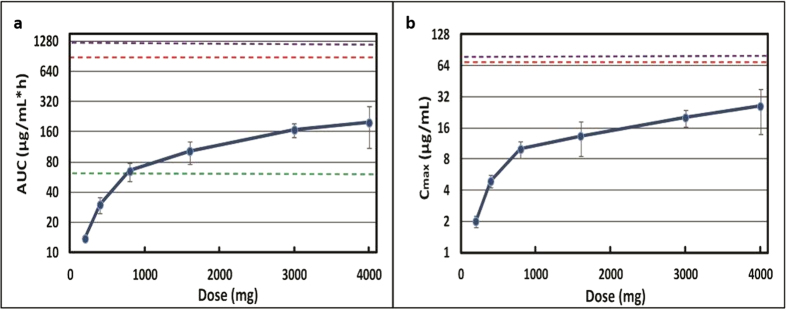
Margins to AUC and C_max_ in humans for ETX0914. (**a**) Human AUC_0-24h_ (±SD) versus dose. The efficacious AUC (horizontal green line) was exceeded at doses >800 mg. The margins to the NOAEL in rat (horizontal magenta line) and dog (horizontal red line) were 6.4- and 4.8-fold, respectively, at the highest 4000 mg dose. (**b**) Human C_max_ (±SD) versus dose. The margins to the NOAEL in rat (horizontal magenta line) and dog (horizontal red line) were 4.9- and 3.7-fold, respectively, at the highest 4000 mg dose.

No serious adverse events (AEs) were encountered during the Phase 1 study. Mild and non-serious AEs entailed transient dysgeusia and headache, which were reversible in all subjects. There were no significant changes in haematological, clinical chemistry, urinalysis, vital signs or ECG. Doses of 2000 mg and 3000 mg were selected for a Phase 2 study in patients with uncomplicated gonococcal infections to maximize the probability of achieving the target ratio of plasma AUC to MIC in more than 90% of the patient population determined via a Monte Carlo simulation for the AUC distribution of ETX0914 (to be published elsewhere). The AUC and C_max_ values for ETX0914 at the various doses also point to a margin of safety as seen by the multiples to the rat and dog NOAEL values (Fig. 5). Hence, ETX0914 holds promise as a first-in-class anti-gonococcal agent and as the first antibacterial drug with a novel mechanism of action since daptomycin. A Phase 2 trial is currently in progress (NCT02257918).

## Methods

### Animals

Mice, rats, dogs and monkeys were maintained under protocols in accordance with the Institutional Animal Care and Use Committee at AstraZeneca and IUCAC guidelines and were approved by in accordance with the American Association for Accreditation of Laboratory Animal Care . Rat and dog toxicology studies were performed in compliance with UK GLP regulations and OECD GLP principles and were approved by the United Kingdom Department of Health.

### Minimum Inhibitory Concentration (MIC)

The bacterial strains included in these studies are maintained in the AstraZeneca Research Collection (ARC). *S. aureus* ARC2381, *S. pneumoniae* ARC548 and ARC 2800, and *N. gonorrhoeae* FA1090, ARC4672, and ARC4680 are contemporary clinical isolates. *S. aureus* ARC3445, ATCC33591-D1 and ATCC33591-D2, *S. pneumoniae* ARC2800, and *N. gonorrhoeae* 49226-TF, ARC4676-D1, ARC4676-D3 and ARC4676-D3-2 were described previously^7^,^9^,^41^. The minimum inhibitory concentration (MIC) against each isolate was determined following the guidelines of the Clinical Laboratory Standards Institute (CLSI). Susceptibility testing against *S. aureus* and *S. pneumoniae* isolates was performed using the broth microdilution method and against *N. gonorrhoeae* isolates using the standard agar dilution method. The quality control isolates obtained from the American Type Culture Collection and used during testing were *N. gonorrhoeae* ATCC49226, *S. aureus* ATCC29213, and *S. pneumoniae* ATCC49619. Reference antimicrobials (ciprofloxacin, levofloxacin, novobiocin, linezolid, gemifloxacin) were obtained from the US Pharmacopeia Convention and MP Biomedicals (Santa Ana, CA), and were tested in accordance with CLSI recommendations. For isolates evaluated in animal efficacy studies, at least ten MIC replicates were performed to obtain the modal MIC. Modal MIC values were used for reporting and in PK/PD analyses of ETX0914 efficacy.

### Isolation and characterization of bacterial isolates resistant to NBTIs (ARC2796)

Suspensions of cultures of methicillin-susceptible *S. aureus* (ARC516) were transferred onto plates of Mueller-Hinton agar containing the NBTI *cis*-4-(2,3-dihydro-1,4-benzodioxin-6-ylmethylamino)-1-[2-(6-methoxy-1,5-naphthyridin-4-yl)ethyl]cyclohexanol^61^ at the following concentrations: 2x, 4x, and 8x MIC. One colony was selected from the 0.12 μg/ml (2xMIC) plate, recovered on drug-free plates, and frozen in glycerol stocks. MIC values were determined. Genomic DNA was prepared from this isolated resistant variant (designated ARC2796), as well as from the original susceptible parent strain, and the genes for the topoisomerase subunits (*gyrA*, *gyrB*, *parC*, and *parE*) were amplified by PCR and sequenced using standard protocols described previously^62^. The genes were found to be identical to ARC516 except for a point mutation found in the *gyrA* gene: Met_121_Lys. This same mutation was isolated by selection in *S. aureus* against NXL101^18^.

### Hollow Fiber Infection Model (HFIM)

Bacterial response over 24 h to various concentrations of ETX0914 was determined using HFIM methods described elsewhere^63^,^64^. Briefly, a 15 mL suspention of *S. aureus* ARC516 (inoculum 10^6^ CFU/mL) grown in cellulosic cartridges were exposed to simulated steady state free plasma drug concentrations of ETX0914. The doses were administered once daily (q24h), every 12 h (q12h), or every 6 h (q6h) to vary the AUC/MIC, C_max_/MIC, and %T > MIC achieved across the cartridges. The system simulated a single-compartment model with mono-exponential elimination. A projected human half-life for both 6 h and 1 h infusion duration was applied in these studies. Serial samples were collected to ascertain simulated drug exposure and bacterial burden over 24 h. A one-compartment linear PK model was fit to the observed concentration-time profiles using WinNonlin 6.3. The change in bacterial burden at 24 h relative to the burden at time zero was correlated to drug exposures determined by the PK model, and a sigmoid E_max_ model was used to analyze the data and simulate the PK/PD indices.

### *S. aureus* infection model and PK

Female CD-1 mice were rendered neutropenic by intraperitoneal injections of cyclophosphamide 150 mg/kg on Day -4 and 100 mg/kg on Day -1 prior to infection. Two hours prior to infection, mice received 50 mg/kg ABT orally. Mice received a second 50 mg/kg ABT administration 12 h later. Thigh infections were produced by intramuscular injection of a mid-log culture of *S. aureus* (ARC516, ATCC33591, USA100 NRS382 or USA300 NRS384) prepared from a fresh subculture, to achieve a target inoculum of 5 × 10^5^ CFU/thigh. ETX0914 was dosed subcutaneously 2 h post-inoculation. Two separate dose response studies were run for each isolate. PK parameters of ETX0914 in infected mice were evaluated following single doses. Blood samples were collected from 3 mice per time point, and a total of 9 time points were collected per dose over 24 h. Plasma was obtained after centrifugation of blood samples in the presence of EDTA and stored at −20 °C until bioanalysis. Concentrations of ETX0914 in mouse plasma were measured by liquid chromatography-mass spectrometry (LC/MS/MS), for which the assay was linear over 0.005–10 μg/mL (r^2^ = 0.995) and quality control accuracy was 100 ± 15%. To reflect free drug, simulated plasma concentration-time data were corrected for ETX0914 protein binding in mouse plasma, 22.1%. PK parameters were determined from single dose data by compartmental analysis (Phoenix WinNonlin 6.3, Certara, St. Louis, MO). Plasma exposures following multiple dose administration in efficacy studies were simulated by mean parameters and verified. The correlation between efficacy and the PK/PD parameter of AUC/MIC was determined using a sigmoidal E_max_ model, and the AUC/MIC magnitudes for different efficacy end points were calculated.

### *In vivo* toxicology

The following dosing formulation for rat and dog 28 day PO studies was used: a 100 mg/mL stock of ETX0914 in 5% dextrose and 2.5M meglumine adjusted to pH 9 using 5M HCl or 2.5M meglumine. The dosing formulations were diluted as required with phosphate buffered saline and dispensed daily. Formulated ETX0914 was administered once daily by oral gavage for 28 days, at dose levels of 200, 500 or 1000 mg/kg/day to groups of 10 male and 10 female Han Wistar rats. A control group of 10 male and 10 females were given the vehicle only. An additional 3 animals per sex were allocated to each group for toxicokinetics wherein blood samples (1 mL) from the tail vein pre-trial and on Day 27 were collected. An additional 5 animals per sex were assigned to the control and high dose groups to demonstrate the reversibility of any findings over a 3 month (91 day) recovery period. Animals were terminated by exposure to CO_2_. The following were assessed: clinical observations, body weight, food consumption, body temperature, hematology, plasma chemistry, toxicokinetics and gross and microscopic pathology. All necropsies consisted of an external and internal examination including body orifices (ear, nostrils mouth, anus and vulva) and cranial, thoracic, abdominal organs and tissues.

Formulated ETX0914 was administered once daily by oral gavage for 28 days, at 100, 200 or 500 mg/kg/day to groups of 3 male and 3 female beagle dogs. A control group of 3 male and 3 females were given the vehicle only. Three animals per sex were assigned to the control and high dose groups to demonstrate the reversibility of any findings over a 92 day recovery period. The electrocardiogram (ECG) of each animal was recorded using a DSI jacketed external telemetry system. Recordings were obtained from all animals once pretrial and on Days 2, 23 and 27 of the dosing period and then in the final week of the recovery period. The following parameters were recorded: PR, QRS, QT and heart rate. An additional recording was obtained from 50 days after the completion of dosing for the females or 51 days after the completion of dosing from the males. The animals were fasted overnight before collecting blood samples (1 mL) from the jugular vein pre-trial and on Day 27. The blood samples were then transferred into tubes containing anticoagulant (0.9 mL 3.8% trisodium citrate). All study animals were terminated by intravenous injection of sodium pentobarbital for necropsy on the day after the final dose was given (day 29). or on day 120 for reversibility studies. All necropsies consisted of an external and internal examination including body orifices (ear, nostrils mouth, anus and vulva) and cranial, thoracic, abdominal organs and tissues.

Samples of the following tissues from both rats and dogs taken at necropsy from all main and recovery study animals were recorded and tissues were weighed, fixed and preserved: Adrenal glands, aortic artery, rib bone marrow smear , brain, epididymides, eyes, femoral bone (including femorotibial joint and distal end and bone marrow), gross lesions/masses, gall bladder, gut associated lymphoid tissues, heart, intestinal duodenum, intestinal ileum, intestinal jejunum, intestinal colon, intestinal caecum, intestinal rectum, kidneys, liver, lacrimal gland, lungs, mandibular lymph node, mandibular, mesenteric lymph node, axiliary lymph node (for dogs), skeletal muscle (from thigh), sciatic nerve, oesophagus, optic nerves, ovaries, pancreas, pituitary gland, prostate gland, mandibular salivary glands, parotid salivary glands, skin and mammary gland, spinal cord (cervical, thoracic and lumbar), spleen, sternum (including bone marrow), stomach, testes, thymus, thyroid glands with parathyroid glands, tongue, trachea, urinary bladder, ureter, uterus with cervix and oviducts, vagina. Bone marrow smears were stained using May-Grunwald-Giemsa as the Romanowsky stain. Modified Davidson’s fixative was used for testes tissues, Davidson’s fixative for eyes and optic nerve tissues and 10% neutral buffered formalin for all other tissues. Wax blocked tissues from all animals were cut into 4–6 μm thick sections and stained with haematoxylin and eosin, and examined by light microscopy. Blood (0.5 mL) for rat haematology analyses was taken via the lateral tail vein and analyzed using a Siemens, ADVIA 120 haematology analyzer.

### Human Phase 1 study

A Phase 1 multipart study^65^ in healthy adult volunteers (informed consent with males and females of non-childbearing potential) with ETX0914 was performed under approval of the AstraZeneca Innovative Medicines Safety Review Board and the United States Food and Drug Administration. ETX0914 was administered orally as an aqueous suspension containing 1% w/v povidone K29–32, 0.2% m/v sodium lauryl sulfate and 0.25% w/v glycerin. In Part A of this study, 48 healthy subjects (ranging in age from 19 to 55 years) under fasting conditions received single ascending doses of 200–4000 mg formulated ETX0914 or placebo (6:2, ETX0914:placebo). In Part B, a second set of 18 healthy subjects (ranging in age from 22 to 48 years) were randomized into two equal dosing cohorts receiving a single dose of either 1500 mg (for 8 subjects) or 3000 mg (for 10 subjects) of ETX0914 in the fasting state and 30 minutes after a meal. Safety monitoring for both Parts A and B included vital signs, electrocardiogram, continuous telemetry, safety laboratory testing (clinical chemistry, haematology and urinalysis) and adverse events monitoring. Blood samples (1 mL) were collected at 0.5, 1, 1.5, 2, 2.5, 3, 4, 5, 6, 8, 10, 12, 24, 36, 48 and 72 h post dose. Urine samples were collected during−12 to 0 hours pre-dose, and 0 to 4, 4 to 8, 8 to 12, 12 to 24, 24 to 48, and 48 to 72 h post-dose. PK parameters were separately determined for the cohorts in each of the Part A and Part B studies. Concentrations of ETX0914 in the plasma and urine were quantified by LC-MS/MS. Plasma PK parameters were calculated using Phoenix WinNonLin software.

## Additional Information

**How to cite this article**: Basarab, G. S. *et al.* Responding to the challenge of untreatable gonorrhea: ETX0914, a first-in-class agent with a distinct mechanism-of-action against bacterial Type II topoisomerases. *Sci. Rep.* **5**, 11827; doi: 10.1038/srep11827 (2015).

## Supplementary Material

Supplementary Information

## Figures and Tables

**Table 1:** MIC versus isolates resistant to ciprofloxacin, the NBTI, novobiocin and ETX0914.

								

Shaded areas represent ≥8-fold increase in MIC relative to susceptible strains; Boldface represents increased sensitivity.

^*a,b*^homologous residues by sequence analysis; ^*c*^homologous to E88 (Fig. 3); ^*d*^homologous to R458 (Fig. 3); ^*d*^sequence aligned with N475 (Fig. 3)^9^; ^e^mutant derived from the spontaneous resistance study with ETX0914.

**Table 2:** Clinical doses and associated fAUC/MIC PK/PD targets in the treatment of complicated skin infection and gonococcal infection.

Compound	Skin infection (*S. aureus*)			Gonococcal infection (*N. gonorrhoeae*)		
	Clinical dose	MIC_90_ (μg/mL)	PK/PD magnitude	Clinical dose	MIC_90_ (μg/mL)	PK/PD magnitude
Ofloxacin	400 mg BID	2	28–40	400 mg UID	0.25–0.5	43–86
Ciprofloxacin	500–750 mg BID	1	19–44	250 mg UID 500 mg UID	0.06–0.12 0.25–0.5	28–56 19–38
ETX0914	800–1600 mg BID	0.25	66–139	800–1600 mg UID	0.12	66–139
