# Corrigendum: Responding to the challenge of untreatable gonorrhea: ETX0914, a first-in-class agent with a distinct mechanism-of-action against bacterial Type II topoisomerases

**DOI:** 10.1038/srep14157

**Published:** 2015-09-18

**Authors:** Gregory S. Basarab, Gunther H. Kern, John McNulty, John P. Mueller, Kenneth Lawrence, Karthick Vishwanathan, Richard A. Alm, Kevin Barvian, Peter Doig, Vincent Galullo, Humphrey Gardner, Madhusudhan Gowravaram, Michael Huband, Amy Kimzey, Marshall Morningstar, Amy Kutschke, Sushmita D. Lahiri, Manos Perros, Renu Singh, Virna J. A. Schuck, Ruben Tommasi, Grant Walkup, Joseph V. Newman

Scientific Reports 5: Article number: 1182710.1038/srep11827; published online: 07142015; updated: 09182015

In this Article, Manos Perros and Ruben Tommasi are incorrectly listed as being affiliated with ‘Shire Pharmaceuticals, 300 Shire Way, Lexington, MA 02421.’ The correct affiliation is listed below:

Entasis Therapeutics, 35 Gatehouse Drive Suite E0, Waltham, MA 02415 USA

In addition, Renu Singh is incorrectly listed as being affiliated with ‘Department of Chemistry, Drug Discovery and Development Center, University of Cape Town, Rondebosch 7701, South Africa.’ The correct affiliation is listed below:

AstraZeneca R&D Boston, Infection iMed, 35 Gatehouse Dr. Waltham, MA 02415 USA

