# Supplementary material for: Responding to the challenge of untreatable gonorrhea: ETX0914, a first-in-class agent with a distinct mechanism-of-action against bacterial Type II topoisomerases
Source: Sci Rep. 2015 Jul 14;5:11827. doi: 10.1038/srep11827 (PMC4501059; doi:10.1038/srep11827)
Supplement: Supplementary Information [file srep11827-s1.doc]

Supplementary Information

Responding to the challenge of untreatable gonorrhea: AZD0914, a first-in-class agent with a distinct mechanism-of-action against bacterial Type II topoisomerases

Gregory S. Basarab, Gunther H. Kern, John McNulty, John P. Mueller, Kenneth Lawrence, Karthick Vishwanathan, Richard A. Alm, Kevin Barvian, Peter Doig, Vincent Galullo, Humphrey Gardner, Madhusudhan Gowravaram, Michael Huband, Amy Kimzey, Marshall Morningstar, Amy Kutschke, Sushmita D. Lahiri, Manos Perros, Renu Singh, Virna J. A. Schuck, Ruben Tommasi, Grant Walkup and Joseph V. Newman

Table of Contents

| Figures S1- S5 | S2-S6 |
| --- | --- |
| Tables S1-S7 | S7-S9 |
| Synthetic methods | S10-S18 |
| Biochemical methods | S18 |
| Pharmacokinetic and distribution determinations | S18-S19 |
| *In vitro t*oxicology | S19 |
| References | S20-S21 |

**Figure S1.** **Key tertiary amino effect reaction to build the spiropyrimidinetrione architecture.** The conversion of chiral *ortho*-morpholinyl aldehydes to chiral final products occurs via the tertiary amino effect reaction involving a sequential Knövenagel condensation, [1,5]-hydride shift and Mannich cyclization. During the course of the reaction, reversible epimerization can occur for the methyl substituent adjacent to the iminium species, and cyclization can occur on either face of the morpholine ring. The 9:1 product mixture reflects the thermodynamic distribution of diastereomers; the major diastereomer is separated from the minor and expresses biologically activity.^1^

**Figure S2.** Comparative haematology analysis in the rat with oral dosing of compound **6** (red) for 5 d, linezolid (orange) for 6 d and AZD0914 (blue) for 28 d. Exposures (AUC) on day 5 for **6** were 950 and 1700 µM*hr at 100 and 250 mg/kg, on day 7 for linezolid were 1900 µM*hr and on day 28 for AZD0914 were 1100±130, 2460±470 and 3820±650 µM*hr (±SD) for the 200, 500 and 1000 mg /kg doses, respectively. The data for **6** represents the average of 2 rats, for linezolid the average of 3 rats and for AZD0914, the average of 9 rats. (a) shows the reticulocytes count from the experiments. The higher dose of **6** and linezolid led to significant suppression. (b) shows lymphocyte counts with the higher dose of **6** leading to suppression. (c) shows neutrophil count with both doses of **6** and linezolid showing suppression. (d) shows the white blood cell count with the higher dose of **6** leading to suppression. AZD0914 fell within statistical equivalence of the vehicle control at the 2 lower doses. There was a statistical elevation in reticulocytes, lymphocytes and white blood cells at the highest dose. Error bars (standard deviations) are shown only above the bar graphs.

**Figure S3. Mg^2+^ concentration effects on re-ligation of *S. aureus* topoisomerase/DNA/inhibitor cleavage complexes** (a) *S. aureus* DNA gyrase ligation assay. Ciprofloxacin-induced cleaved DNA is ligated upon removal of Mg^2+^ by EDTA. EDTA addition results in free Mg^2+^ concentrations between 0.06 μM and 8 mM as indicated on the x-axis. No ligation is observed for cleaved DNA induced by AZD0914 over the entire range tested. (b) *S. aureus* Topo IV ligation assay. Ciprofloxacin-induced cleaved DNA is ligated upon removal of free Mg^2+^ by addition of EDTA. EDTA addition results in free magnesium concentrations between 0.06 μM and 8 mM as indicated on the x-axis. No ligation is observed for cleaved DNA induced by AZD0914 over the entire range of EDTA tested. The last lane on each gel marked ′a′ represents a control reaction without inhibitor.


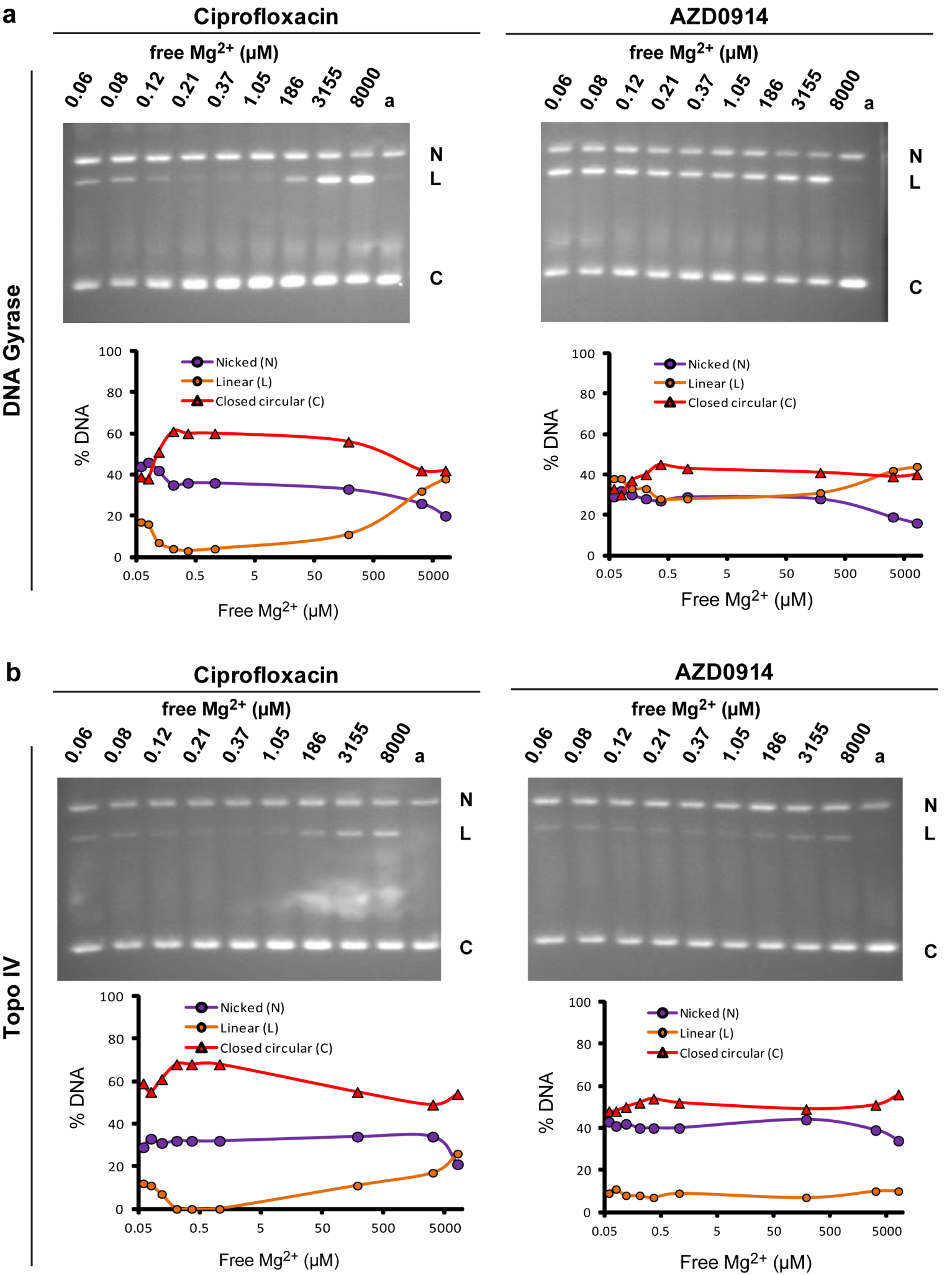


**Figure S4.** Representative autoradiograms show rapid (5 minutes) distribution of radioactivity (dark areas) into vascularized tissue after a single IV infusion of ^14^C-AZD0914 to male Lister Hood rats. The dark shade for the liver tissue indicates a higher drug accumulation. The light area for the brain shows little CNS penetration. No significant penetration into vascularized eye and testis tissue is noted.

**Figure S5. Time course for dosing AZD0914 in humans.** Geometric mean plasma concentration-time profiles of AZD0914 following single oral doses of 200 mg to 4000 mg, from 0 to 72 hours post-dose in humans. C_max_ and AUC_0-14h_ increased as the dose was escalated. Variability in exposures in individual patients varied from 12% at the lowest dose to 46% at the highest dose. Error bars represent the standard deviation (SD).


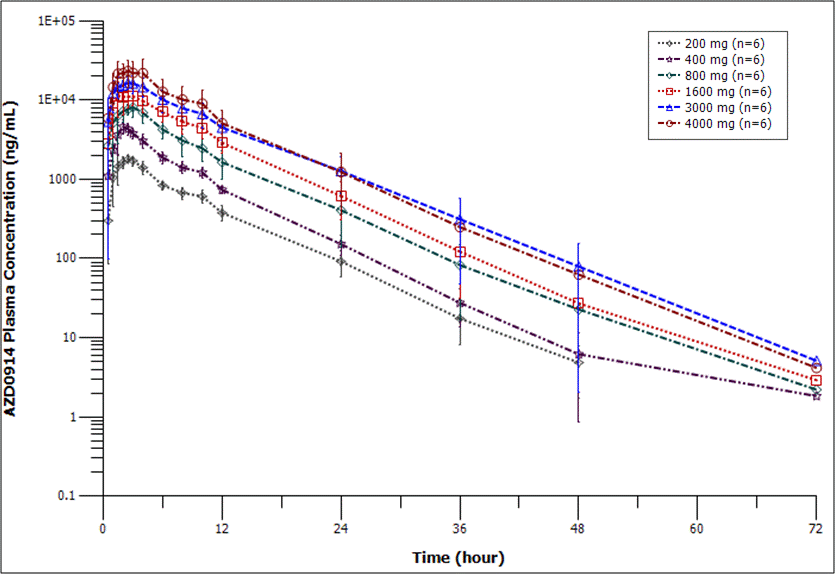


| **Table S1:** AZD0914 *in vitro* antibacterial activity | | | | |
| --- | --- | --- | --- | --- |
| Indication | Organism | # tested | MIC Range (µg/ml) | MIC_90_ (µg/ml) |
| Skin and skin structure infections | *Staphylococcus aureus* | 11640 | ≤ 0.008-0.5 | 0.25^2^ |
|  | *Streptococcus pyogenes* | 1358 | ≤ 0.015-0.5 | 0.25^2^ |
|  | Coagulase-negative Staphylococci | 1923 | 0.015-2.0 | 0.25^2^ |
|  | *Streptococcus agalactiae* | 441 | 0.03-0.5 | 0.25^2^ |
| Respiratory tract infections | *Streptococcus pneumoniae* | 2324 | ≤ 0.015-0.5 | 0.25^2^ |
|  | *Moraxella catarrhalis* | 145 | 0.015-0.5 | 0.25^2^ |
|  | *Haemophilus influenzae* | 352 | 0.03-2.0 | 0.5^2^ |
|  | *Mycoplasma pneumoniae* | 21 | 0.5-1.0 | 1.0^3^ |
| Sexually transmitted diseases | *Neisseria gonorrhoeae* | 473 | 0.004 to 0.25 | 0.125^4-6^ |
|  | *Chlamydia trachomatis* | 10 | 0.06-0.5 | 0.25^3^ |
|  | *Mycoplasma genitalium* | 11 | 0.5-1.0 | 1.0^3^ |
|  | *Ureaplasma* spp. | 21 | 0.125-2 | 1.0^3^ |

| **Table S2:** *In vitro* ion channel function IC_50_ (µM) | | | |  |  |  |
| --- | --- | --- | --- | --- | --- | --- |
|  |  | **Compound** | | | | |
| **Channel** | **Function/Effects** | **1** | **5** | **6** | **7** | **AZD0914** |
| hERG^7^ | K^+^ rectifying current/QT interval | 232 | >100 | 237 | 91 | 316 |
| hNav1.5^8^ | Initiation of cardiac action potential/QRS interval | >316 | >80 | >333 | >100 | >333 |
| hCav1.2^9^ | Ca^2+^ entry into excitable cells/hypotension, decreased contractility/ PR interval | >100 | >100 | >100 | >100 | 326 |
| Iks/Kv7.1^10^ | Slowly activating K^+^ delayed rectifier/late repolarization/QT interval | >316 | >100 | >333 | >100 | >333 |
| Ikr/Kv1.5^8^ | Rapidly activating K^+^ delayed rectifier/QT interval | >100 | >100 | NT | >100 | >333 |
| Ito/Kv4.3^11^ | Transient outward K^+^ early repolarization/ QT interval | >292 | >100 | 224 | >90 | 216 |

| **Table S3** Single dose AZD0914 pharmacokinetic parameters in preclinical species | | | | |
| --- | --- | --- | --- | --- |
| Parameters | CD-1 mouse (female) | Han-Wister rat (male) | Beagle dog (male) | Cynomologus monkey (male) |
| Dose: IV/PO (mg/kg) | 3 / 10 | 10 / 10 | 2.5 / 5 | 2.5 / 5 |
| Cl_p_ (mL/min/kg. IV) | 70 | 22 | 4 | 12 |
| V_ss_ (L/kg, IV) | 3.6 | 1.2 | 0.8 | 1.3 |
| T_1/2_ (h, IV) | 0.5 | 0.7 | 3.0 | 1.6 |
| C_max_ (µM, PO) | 1.7 | 1.7 | 7.1 | 6.5 |
| T_max_ (h, PO) | 1.6 | 1.8 | 3.1 | 1.6 |
| T_1/2_ (h, PO) | 1.6 | 1.8 | 3.1 | 1.6 |
| Ka (h^-1^) | 1.7 | 1.6 | 1.2 | - |
| %F | 46 | 34 | 71 | 58 |
| PPB (%free)^a^ | 22.1±0.3 | 19.1±1.1 | 18.4±0.3 | ND^c^ |
| CL_int_ (µL/min/10^6^ cells)^b^ | 7.0 | 10 | 3.8 | ND |
| ^a^*in vitro* at AZD0914 concentration of 1-50 µM; ^b^*in vitro* hepatocytes at AZD0914 concentration of 1 µM; ^c^not determined | | | | |

| Table S4. Total radioactivity (nmol/g) of ^14^C-AZD0914 in selected tissues following 15 min intravenous infusion (25 mg/kg) to male pigmented rats | | | | | |
| --- | --- | --- | --- | --- | --- |
| Tissue | Time after AZD0914 administration | | | | |
|  | 5 minutes | 1 hour | 4 hours | 24 hours | 48 hours |
| Adrenal cortex | 84 | 40 | 8.7 | NI^e^ | NI |
| Adrenal medulla | 43 | 26 | 4.5 | NI | NI |
| Aortic wall | 53 | 58 | 13 | NI | NI |
| Bile*^a^* | 1200 | 1500 | OE*^c^* | NI | NI |
| Bone marrow*^b^* | 35 | 20 | 4.0 | NI | NI |
| Brain | 0.79 | 0.72 | BLQ*^d^* | NI | NI |
| Choroid & retinal pigment epithelium | 16 | 17 | 8.1 | NI | NI |
| Heart blood | 27 | 18 | 4.2 | NI | NI |
| Liver | 320 | 190 | 69 | 0.35 | BLQ |
| Lung | 52 | 31 | 6.9 | NI | NI |
| Lymph node | 29 | 23 | 4.1 | NI | NI |
| Myocardium | 73 | 42 | 6.9 | NI | NI |
| Renal cortex*^b^* | 170 | 130 | 39 | NI | NI |
| Renal medulla (inner)*^b^* | 70 | 32 | 8.0 | NI | NI |
| Renal medulla (outer)*^b^* | 68 | 120 | 18 | NI | NI |
| Skeletal muscle | 29 | 20 | 4.2 | NI | NI |
| Skin, pigmented | 15 | 20 | 3.6 | NI | NI |
| Spinal cord | 1.0 | 1.0 | 0.30 | NI | NI |
| Testis | 2.4 | 5.7 | 2.6 | NI | NI |
| Urine*^a^* | 51 | 400 | 100 | NI | NI |
| White fat | 6.6 | 8.9 | NI | NI | NI |
| Limit of quantification | 0.30 | 0.33 | 0.24 | 0.32 | 0.24 |

*^a^*limit of quantification for this organ = 0.23 nmol/g; ^b^limit of quantification for this organ = 0.27 nmol/g;

*^c^*overexposed; *^d^*below limit of quantification; *^e^*not identified for quantification

| Table S5 Calculated fAUC/MIC parameters for AZD0914 versus *S. aureus* isolates in the neutropenic mouse thigh model. Values are represented as Mean ± Standard error of the Mean. | | | |
| --- | --- | --- | --- |
| Isolate | Modal MIC (µg/mL) | Stasis | 1-log reduction |
| MSSA ARC516 | 0.0625 | 98 ± 17 | 245 ± 81 |
| MRSA ATCC33591 | 0.125 | 43 ± 5 | 96 ± 35 |
| USA100 NRS382 | 0.125 | 43 ± 5 | 83 ± 23 |
| USA300 NRS384 | 0.25 | 80 ± 5 | 132 ± 16 |

| Table S6 Comparison of preclinical and clinical complicated skin and skin structure infections (cSSTI) PK/PD magnitudes associated with efficacy against *S. aureus*. Preclinical model magnitudes for Linezolid and Levofloxacin are represented as Mean ± Standard error of the Mean. | | | | | |
| --- | --- | --- | --- | --- | --- |
| Compound | PK/PD index | Clinical dose (mg/day) | PK/PD magnitude | PK/PD magnitude associated with preclinical model | |
|  |  |  |  | Measured | Literature |
| Linezolid | fAUC/MIC | 1200 | 70 | Stasis: 66 ± 25 | Stasis: 66 ± 6 |
| Levofloxacin | fAUC/MIC | 500 | 28 | Stasis: 22 ± 2  1-log: 45 ± 15 | Stasis: 29 ± 4  1-log: 62 ± 15 |
| Ceftriaxone | %T>MIC | 2000 | 21% | ND | Stasis: 25%  1-log: 40% |

| **Table S7:** AZD0914 *in vivo* effects and plasma exposure in rat and dog | | | | |
| --- | --- | --- | --- | --- |
| **Study** | **Salient Pathology Findings** | **Dose (mg/kg/day)** | **AUC (µg*h/mL) (±SD)** | **C_max_ (µg/mL)**  **(±SD)** |
| 1 month rat (po) | NOAEL | 200 | 1100±130 | 110±30 |
|  | Reduced activity, pilo-erection, irregular respiration  Seminal vesicle: secretory depletion, atrophy  Epididymis: cellular debris (minimal)  Spleen: increased congestion/haematopoiesis  Cecum & rectum: mucosal hyperplasia | 500 | 2460±470 | 170±30 |
|  | As above & testes tubular degeneration partially reversible 3 months post dosing | 1000 | 3820±650 | 260±55 |
| 1 month dog (po) | NOAEL | 100 | 870±240 | 90±22 |
|  | Testes tubular degeneration partially reversible 3 months post dosing | 200 | 2160±460 | 210±18 |
|  | As above plus:  Epididymis cellular debris  Caecum/illium/rectum necrosis | 500 | 2780±1140 | 220±94 |
| CV dog telemetry(single dose, iv) | NOEL | 50 | --- | 63 |
|  | Emesis  Increase in cardiac contractility\relaxation and heart rate  Decrease in systolic & diastoloic blood pressure  Orthostatic hypertension | 100 | --- | 160 |

**Synthetic Methods**

**General Considerations** All of the solvents and reagents used were obtained commercially and used as such unless noted otherwise. ^1^H NMR spectra were recorded in DMSO-*d_6_* solutions at 300 K using a Bruker Ultrashield 300 MHz instrument, a Bruker Ultrashield 400 MHz instrument or a Bruker Ultrashield 600 MHz instrument. ^13^C NMR spectra were recorded in DMSO-*d_6_* solutions at 300 K and at 101 MHz using a Bruker Ultrashield 400 MHz instrument. ^19^F NMR spectra were recorded at 282 MHz at 300 K using a Bruker Ultrashield 300 MHz instrument. Chemical shifts are reported as parts per million relative to TMS (0.00) for ^1^H and ^13^C NMR and CFCl_3_ for ^19^F NMR. High-resolution mass spectra (HRMS) were obtained using a hybrid quadrupole time-of-flight mass spectrometer (microTOFq II, Bruker Daltonics) in ESI^+^ mode. Silica gel chromatographies were performed on an ISCO Combiflash Companion Instruments using ISCO RediSep Flash Cartridges (particle size: 35-70 microns) or Silicycle SiliaSep Flash Cartridges (particle size: 40-63 microns. All final compounds were determined to be greater than 95% pure via analysis by reversed phase UPLC-MS (retention times, RT, in minutes) with a Waters Acquity UPLC instrument with DAD and ELSD and a UPLC HSS T3, 2.1 x 30 mm, 1.8 um column and a gradient of 2 to 98% acetonitrile in water with 0.1% formic acid over 2.0 minutes at 1 mL/min. Injection volume was 1 µL and the column temperature was 30 °C. Detection was based on electrospray ionization (ESI) in positive and negative polarity using Waters ZQ mass spectrometer (Milford, MA, USA), diode-array UV detector from 210 to 400 nm, and evaporative light scattering detector (Sedex 75, Sedere, Alfortville Cedex, France). Optical rotations were obtained on a Jasco P-2000 instrument using a Na light source at 589 nM and a Dichrom polarizer. When not indicated, compound intermediates and reagents were purchased from chemical supply houses.

**(4*R*)-3-{6-[(2*R*,6*R*)-2,6-Dimethylmorpholin-4-yl]-5-(1,3-dioxolan-2-yl)-7-fluoro-1,2-benzoxazol-3-yl}-4-methyl-1,3-oxazolidin-2-one** A solution of 11.9 g (118 mmol) of (*S*)-4-methyloxazolidin-2-one^12^ in 20 mL DMF was added over 10 min to a stirred suspension of NaH (60% oil dispersion, 4.71 g, 117 mmol) in 140 mL DMF at 0 °C. The mixture was stirred at rt for 90 min, and a solution of (42.5 g, 117 mmol) of 3-chloro-6-((2R,6R)-2,6-dimethylmorpholino)-5-(1,3-dioxolan-2-yl)-7-fluorobenzo[d]isoxazole^13^ in 30 mL DMF was added. This mixture was heated at 90 °C for 4 h, cooled to rt and quenched with saturated aqueous NH_4_Cl. Solvents were removed and the residue was partitioned between water and EtOAc. The organic layer was separated, washed with brine, dried (Na_2_SO_4_) and concentrated. The residue was chromatographed on silica gel (20-40% EtOAc gradient in CHCl_3_) to afford 17.5 g starting material and 14.6 g (29%) of the title compound: ^1^H NMR (300 MHz, DMSO-*d_6_*) δ 8.23 (s, 1H), 6.18 (s, 1H), 4.66-4.84 (m, 2H), 4.18-4.30 (m, 1H), 3.91-4.14 (m, 6H), 3.22 (d, *J*=10.74 Hz, 2H), 2.89 (dd, *J*=5.27, 10.74 Hz, 2H), 1.43 (d, *J*=6.03 Hz, 3H), 1.20-1.30 (m, 6H); ^19^F NMR (282 MHz, DMSO-*d_6_*) δ -145.43 : MS (ES) MH^+^: 422.3 for C_20_H_24_FN_3_O_6_.

**(2*R*,4*S*,4a*S*)-11-Fluoro-2,4-dimethyl-8-[(4*S*)-4-methyl-2-oxo-1,3-oxazolidin-3-yl]-1,2,4,4a-tetrahydro-2'*H*,6*H*-spiro[1,4-oxazino[4,3-*a*][1,2]oxazolo[4,5-*g*]quinoline-5,5'-pyrimidine]-2',4',6'(1'*H*,3'*H*)-trione** (AZD0914) A mixture of the preceding compound (585 mg, 1.39 mmol) and barbituric acid (178 mg, 1.39 mmol) in 8 mL acetic acid and 2 mL water was heated at 110 °C for 3.5 h. The solvents were removed, and the residue was purified by Supercritical Fluid Chromatography (Chiralpak IC column with 30% MeOH and 70% CO_2_ mobile phase, 40˚ C, 100 bar, 40 mL/min flow rate) to afford 480 mg (71%) of the title compound as the major eluting component. ^1^H NMR (400MHz, DMSO-d_6_) δ = 11.81 (br. s., 1H), 11.45 (br. s., 1H), 7.59 (s, 1H), 4.69 (t, *J*=7.8 Hz, 1H), 4.67 - 4.59 (m, 1H), 4.18 (dd, *J*=4.9, 7.7 Hz, 1H), 4.10 (d, *J*=12.5 Hz, 1H), 3.94 (d, *J*=8.8 Hz, 1H), 3.84 - 3.74 (m, 1H), 3.72 - 3.59 (m, 2H), 3.18 - 3.06 (m, 1H), 2.94 (d, *J*=14.1 Hz, 1H), 1.42 (d, *J*=5.8 Hz, 3H), 1.15 (d, *J*=6.3 Hz, 3H), 0.89 (d, *J*=6.3 Hz, 3H); ^19^F NMR (282 MHz, DMSO-*d_6_*) δ -158.16; ^13^C NMR (75 MHz, DMSO-*d_6_*) δ 170.9, 167.6, 153.6 (d, *J_CF_*=12.7 Hz), 153.6, 152.8 (d, *J_CF_*=2.2 Hz), 149.4, 134.7 (d, *J_CF_*=1.7 Hz), 133.2 (d, *J_CF_*=238.8 Hz), 122.2 (d, *J_CF_*=2.2 Hz), 118.5, 106.2, 72.4, 72.1, 71.6, 64.4, 56.2 (d, *J_CF_*=9.9 Hz), 52.9, 51.0, 38.6, 19.9, 18.1, 18.1; UPLC RT = 0.92 min, (ES) MH^+^: 488.1 for C_22_H_23_FN_5_O_7_; HRMS (ES) MH^+^ calcd for C_22_H_23_FN_5_O_7_ 488.1576 found 488.1580. [α]_D_^20^ = -188 (c = 1; MeOH).

**(2*R*,4*S*,4a*S*)-11-Fluoro-2,4-dimethyl-8-[(4*S*)-4-methyl-2-oxo-1,3-oxazolidin-3-yl]-1,2,4,4a-tetrahydro-2'*H*,6*H*-spiro[1,4-oxazino[4,3-*a*][1,2]oxazolo[4,5-*g*]quinoline-5,5'-[5-^14^C]pyrimidine]-2',4',6'(1'*H*,3'*H*)-trione** (^14^C-AZD0914) A mixture of 6-((2*R*,6*R*)-2,6-dimethylmorpholino)-7-fluoro-3-((S)-4-methyl-2-oxooxazolidin-3-yl)benzo[d]isoxazole-5-carbaldehyde^13^ (428 mg, 1.13 mmol) and (5-^14^C)-barbituric acid^14^ (162 mg, 1.25 mmol) in 10 mL acetic acid and 2 mL water was heated at 110 °C for 3 h. The solvents were removed, and the residue was taken up in toluene 3 times and solvents were removed. The residue was chromatographed on silica gel (0-30% EtOAc gradient in CH_2_Cl_2_) to afforded 406 mg (73%) of the title compound as the major eluting component. LC MS (ES) MH^+^: 492.45 for C_20_^13^C_2_H_22_FN_3_^15^N_2_O_7_; ^1^H NMR and ^19^F NMR are identical to those for unlabelled AZD0914. Specific Activity: 55.6 mCi/mmol (2060 MBq/mmol) incorporation: 0.89 labelled atoms / molecule based on 100:12.4 MH^+^ 490:488 ratio; Molecular weight at this incorporation: 489.2. The material was diluted 10-fold with unlabelled AZD0914 for *in vitro* and *in vivo* experiments.

**4,6-^13^C_2_-1,3-^15^N-2,4,6(1*H*,3*H*,5*H*)-Pyrimidinetrione**. A solution of NaOEt (2.383 mL, 6.38 mmol) in EtOH ethanol (0.6 mL) was added to ^15^N_2_-urea (360 mg, 5.80 mmol) and the resulting slurry heated to 50 °C until the urea dissolved (1h ). (1,3-^13^C_2_)-diethylmalonate (1.01 g, 6.21 mmol) was added and the mixture heated at 110 °C for 2 h in a microwave reactor to give a wet, white solid. The crude solid was transfered to 20 mL water and heated to 50 °C. The stirred suspension was acidified to pH 1 by the addition of conc. HCl (0.75 mL) to give a clear, colourless solution that was cooled to 4 °C and held at this temperature overnight. The crystaline product was collected by filtration, washed with water and dried under reduced pressure to give the title compound (622 mg, 4.71 mmol, 81 %) as pale yellow needles.

**(2*R*,4*S*,4a*S*)-11-Fluoro-2,4-dimethyl-8-[(4*S*)-4-methyl-2-oxo-1,3-oxazolidin-3-yl]-1,2,4,4a-tetrahydro-2'*H*,6*H*-spiro[1,4-oxazino[4,3-*a*][1,2]oxazolo[4,5-*g*]quinoline-5,5'-[1',3'-15N_2_,4',6'-^13^C_2_]pyrimidine]-2',4',6'(1'*H*,3'*H*)-trione** (^14^C_2_^15^N_2_-AZD0914) A mixture of 6-((2*R*,6*R*)-2,6-dimethylmorpholino)-7-fluoro-3-((S)-4-methyl-2-oxooxazolidin-3-yl)benzo[d]isoxazole-5-carbaldehyde^13^ (455 mg, 1.13 mmol) and the previous compound (181 mg, 1.45 mmol) in 10 mL acetic acid and 2 mL water was heated at 110 °C for 3.5 h. The solvents were removed, and the residue was taken up in toluene 3 times and solvents were removed. The residue was chromatographed on silica gel (0-30% EtOAc gradient in CH_2_Cl_2_) to afforded 455 mg (77%) of the title compound as the major eluting component. The material was used as an internal standard for pharmacokinetic analyses. LC MS (ES) MH^+^: 492.45 for C_20_^13^C_2_H_22_FN_3_^15^N_2_O_7_. ^1^H NMR (600 MHz, DMSO-*d_6_*) 11.8 (br. d, *J*=90 Hz, 1H), 11.4 (br. s., *J*=90 Hz, 1H), 7.6 (s, 1H), 4.69 (t, *J*=7.8 Hz, 1H), 4.6 - 4.7 (m, 1H), 4.18 (dd, *J*=4.9, 7.7 Hz, 1H), 4.10 (d, *J*=12.5 Hz, 1H), 3.94 (d, *J*=8.8 Hz, 1H), 3.84 - 3.74 (m, 1H), 3.7 - 3.64 (m, 1H), 3.62 (d, *J*=12.5, 1H) 3.18 - 3.06 (m, 1H), 2.94 (d, *J*=14.1 Hz, 1H), 1.42 (d, *J*=5.8 Hz, 3H), 1.15 (d, *J*=6.3 Hz, 3H), 0.89 (d, *J*=6.3 Hz, 3H).

**(2*R*,4*S*,4a*S*)-11-Fluoro-2,4,8-trimethyl-2,4,4a,6-tetrahydro-1*H*,1'*H*-spiro[isoxazolo[4,5-g][1,4]oxazino[4,3-a]quinoline-5,5'-pyrimidine]-2',4',6'(3'*H*)-trione** (**5**) was prepared as described^1^.

**3-Chloro-5-(dimethoxymethyl)-6-((2*R*,6*R*)-2,6-dimethylmorpholino)-7-fluorobenzo[d]isoxazole** A solution of 3-chloro-6-((2*R*,6*R*)-2,6-dimethylmorpholino)-7-fluorobenzo[d]isoxazole-5-carbaldehyde^15^ (18.1 g, 57.9 mmol), 2,2-dimethoxypropane (72.0 ml, 579 mmol), and *p*-TsOH hydrate (0.110 g, 0.58 mmol) was stirred at rt overnight. After quenching with saturated aq. NaHSO_3_, the mixture was extracted with EtOAc, which was washed with brine. Drying (Na_2_CO_3_) of the organic extract and removal of solvent gave 18.8 g (100%) of the title compound as a yellow solid. ^1^H NMR (300 MHz, DMSO-*d_6_*) δ 7.62 (s, 1H), 5.77 (s, 1H), 4.10 (dt, *J*=3.40, 5.95 Hz, 2H), 3.33 (s, 3H), 3.29 (s, 3H), 3.22 (d, *J*=11.3 Hz, 2H), 2.85 (d, *J*=5.7 Hz, 1H), 2.81 (d, *J*=5.7 Hz, 1H), 1.25 (d, *J*=5.85 Hz, 6H); ^19^F NMR (282 MHz, DMSO-*d_6_*) δ -142.80; LCMS (ES) (M-MeO^-^)^+^: 327.0 for C_16_H_20_ClFN_2_O_4_.

**5-(Dimethoxymethyl)-6-((2*R*,6*R*)-2,6-dimethylmorpholino)-7-fluoro-3-(1*H*-1,2,4-triazol-1-yl)benzo[d]isoxazole** NaH (60% dispersion, 2.01 g, 83.6 mmol) was added to a solution of 1,2,4-triazole (5.78 g, 83.6 mmol) in 120 mL DMF at rt. Gas evolution and a slight exotherm were observed. After the resulting slurry was stirred for 20 min, 10 g (27.9 mmol) of the preceding compound in 60 mL DMF was added, and the mixture was heated at 60 ˚C for 24 h. After cooling to rt and quenching with saturated aq. NH_4_Cl, the mixture was extracted twice with EtOAc, which was washed with brine. Drying (Na_2_SO_3_) of the organic extract and removal of solvent gave a residue that was chromatographed on silica gel (20% EtOAc in hexanes) to afford 8.05 g (74%) of the title compound as an off-white solid. ^1^H NMR (300 MHz, DMSO-*d_6_*) δ 9.54 (s, 1H), 8.60 (s, 1H), 5.79 (s, 1H), 4.11 (dt, *J*=3.2, 6.0 Hz, 2H), 3.34 (s, 3H), 3.30 (s, 3H), 3.18-3.28 (m, 2H), 2.86-2.93 (m, 1H), 2.84 (d, *J*=5.5 Hz, 1H), 1.26 (d, *J*=5.85 Hz, 6H); ^19^F NMR (282 MHz, DMSO-*d_6_*) δ -143.51; LCMS (ES) MH^+^: 392.1 for C_18_H_22_FN_5_O_4_.

**(2*R*,4*S*,4a*S*)-11-Fluoro-2,4-dimethyl-8-(1*H*-1,2,4-triazol-1-yl)-2,4,4a,6-tetrahydro-1*H*,1'*H*-spiro[isoxazolo[4,5-g][1,4]oxazino[4,3-a]quinoline-5,5'-pyrimidine]-2',4',6'(3'*H*)-trione** (**6**) A mixture of the preceding compound (15.1 g, 1.39 mmol) and barbituric acid (178 mg, 1.39 mmol) in 8 mL acetic acid and 2 mL water was heated at 110 °C for 3.5 h. The solvents were removed, and the residue was purified using Supercritical Fluid Chromatography (Chiralpak IC column with 30% MeOH and 70% CO_2_ mobile phase, 40˚ C, 100 bar, 40 mL/min flow rate) to afford 480 mg (71%) of the title compound as the major eluting component. ^1^H NMR (300 MHz, DMSO-*d_6_*) δ 11.73 (s, 1H), 11.40 (s, 1H), 9.16 (s, 1H), 8.22-8.28 (m, 1H), 7.53 (s, 1H), 4.13 (d, *J*=12.8 Hz, 1H), 4.00 (d, J=8.9 Hz, 1H), 3.78-3.91 (m, 1H), 3.61-3.77 (m, 1H), 3.45 (d, *J*=14.2 Hz, 1H), 3.05-3.21 (m, 1H), 3.00 (d, *J*=13.97 Hz, 1H), 1.16 (d, *J*=6.0 Hz, 3H), 0.92 (d, *J*=6.4 Hz, 3H); ^19^F NMR (282 MHz, DMSO-*d_6_*) δ -157.06; ^13^C NMR (101 MHz, DMSO-*d_6_*) δ 170.8, 167.6, 153.8, 153.6 (d, *J_CF_*=13.2 Hz), 151.4 (d, *J_CF_*=2.2 Hz), 149.4, 144.3, 135.9, 133.3 (d, *J_CF_*=240.8 Hz), 125.0 (d, *J_CF_*=1.5 Hz), 115.9, 105.5, 72.1, 71.7, 64.5, 56.3 (d, *J_CF_*=8.8 Hz), 52.8, 38.2, 18.2, 18.1; UPLC RT = 0.88 min, (ES) MH^+^: 455.9 for C_20_H_19_FN_7_O_5_; HRMS (ES) MH^+^ calcd for C_20_H_19_FN_7_O_5_ 456.1426 found 456.1424. [α]_D_^20^ = -210 (c = 0.1; MeOH).

**Ethyl 6,7-difluoro-5-formylbenzo[d]isoxazole-3-carboxylate** A solution of ethyl 5-(dimethoxymethyl)-6,7-difluorobenzo[d]isoxazole-3-carboxylate^15^ (19.4 g, 64.4 mmol) in 50 mL AcOH was heated at 100 °C for 20 h. The reaction mixture was concentrated and the residue was taken up in EtOAc, which was washed 3 times with saturated aqueous NaHCO_3_ and once with brine. The combined aqueous layers were back-extracted with twice more with EtOAc. The organic layers were combined, dried (MgSO_4_) and concentrated to give the title compound an an oil (15.0 g, 58.8 mmol, 91 %). ^1^H NMR (300 MHz, DMSO-*d_6_*) δ 10.25 (s, 1H), 8.35 (dd, *J*=1.7, 5.65 Hz, 1H), 4.53 (q, *J*=7.2 Hz, 2H), 1.42 (t, *J*=7.1 Hz, 3H); ^19^F NMR (282 MHz, DMSO-*d_6_*) δ -142.34 (d), -158.14 (d); LCMS (ES) MH^+^: 256.1 for C_11_H_7_F_2_NO_4_.

**Ethyl 6-((2*R*,6*R*)-2,6-dimethylmorpholino)-7-fluoro-5-formylbenzo[d]isoxazole-3-carboxylate** A mixture of the preceding compound (15 g, 58.8 mmol), diisopropylethylamine (12.3 mL, 70.5 mmol) and (2*R*,6*R*)-2,6-dimethylmorpholine (8.09 mL, 64.66 mmol) in 200 mL CH_3_CN was heated at 80 °C for 28 h. Solvent was removed and the residual oil was chromatographed on silica gel (hexanes/EtOAc gradient) to afford the title compound as an oil (16 g, 45.7 mmol, 78 %). ^1^H NMR (300 MHz, DMSO-*d_6_*) δ 10.35 (s, 1H), 8.17 (d, *J*=0.75 Hz, 1H), 4.51 (q, *J*=7.1 Hz, 2H), 4.15 (dt, *J*=3.3, 6.1 Hz, 2H), 3.36-3.45 (m, 2H), 2.96-3.13 (m, 2H), 1.40 (t, *J*=7.1 Hz, 3H), 1.22 (d, *J*=6.6 Hz, 6H); ^19^F NMR (282 MHz, DMSO-*d_6_*) δ -144.66; LCMS (ES) MH^+^: 351.1 for C_17_H_19_FN_2_O_5_.

**Ethyl 5-(dimethoxymethyl)-6-((2*R*,6*R*)-2,6-dimethylmorpholino)-7-fluorobenzo[d]isoxazole-3-carboxylate** A solution of the preceding compound (45.2 g, 129.02 mmol) and *p*-TsOH (1.23 g, 6.45 mmol) in 150 mL 2,2-dimethoxypropane was stirred at rt for 24 h. The reaction mixture was concentrated, and the residue was diluted with EtOAc, which was washed 3x with saturated aqueous NaHCO_3_. Drying (MgSO_4_) and removal of solvent gave an oil that chromatographed on silica gel (hexanes/EtOAc gradient) to afford the title compound (42.8 g, 108 mmol, 84 %) as an oil. ^1^H NMR (300 MHz, DMSO-*d_6_*) δ ^1^H NMR (300 MHz, DMSO*_d6_*) δ 7.94 (s, 1H), 5.78 (s, 1H), 4.50 (q, *J*=7.10 Hz, 2H), 4.06-4.18 (m, 2H), 3.34 (s, 3H), 3.29-3.31 (m, 3H), 3.22 (d, *J*=9.23 Hz, 2H), 2.84 (dd, *J*=5.5, 11.1 Hz, 2H), 1.39 (t, *J*=7.1 Hz, 3H), 1.20-1.3 (br s, 6H); ^19^F NMR (282 MHz, DMSO-*d_6_*) δ -142.68LCMS (ES) MH^+^: 397.1 for C_19_H_25_FN_2_O_6_.

**6-((2*R*,6*R*)-2,6-Dimethylmorpholino)-7-fluoro-5-formyl-*N*-(2,2,2-trifluoroethyl)benzo[d]isoxazole-3-carboxamide** Me_3_Al (2N in toluene, 12.3 ml, 24.6 mmol) was added slowly (gas evolution) over 10 min to a mixture of 2,2,2-trifluoroethanamine (2.34 g, 17.2 mmol) suspended in 100 ml of toluene cooled to 0 °C. After warming to rt and stirring for 1.5 h, 2.5 g (6.31 mmol) of the preceding compound was added, and the reaction was heated at 40 °C for 20 h. The reaction was cooled in an ice bath, and 5 ml of 1N HCl was added dropwise (vigorous gas evolution) over 20 min. Stirring was continued at rt for 3 h. Solids were filtered off, and the filtrate was washed with 1N HCl and saturated brine. The organic layer was dried (MgSO_4_) and concentrated to give an oil that was chromatographed on silica gel (hexanes-EtOAc gradient) to afford the title compound (2.54 g, 100 %). The material was used in the next step without further purification. ^1^H NMR (300 MHz, DMSO-*d_6_*) δ 10.3 (s, 1H), 9.9 (q, *J*=6.5 Hz, 1H), 8.2 (s, 1H), 4.1 (m, 4H), 3.4 (d, 1H), 3.0 (m, 2H), 1.2 (m, 6H); ^19^F NMR (282 MHz, DMSO-*d_6_*) δ -70.13, -144.58; LCMS (ES) MH^+^: 404.1 for C_17_H_17_F_4_N_3_O_4_.

**(2*R*,4*R*,4a*R*)-11-Fluoro-2,4-dimethyl-2',4',6'-trioxo-*N*-(2,2,2-trifluoroethyl)-2,2',3',4,4a,4',6,6'-octahydro-1*H*,1'*H*-spiro[isoxazolo[4,5-g][1,4]oxazino[4,3-a]quinoline-5,5'-pyrimidine]-8-carboxamide** (**7**) The preceding compound (2.27 g, 5.63 mmol) and barbituric acid (0.79 g, 6.19 mmol) in 20 mL of 20% water in acetic acid was heated at 110 °C for 4 h. The mixture was concentrated to an oil, which was dissolved in CH_2_Cl_2_ and methanol and absorbed onto Celite for purification on silica gel (gradient of CH_2_Cl_2_/EtOAc). The material was further purified by Supercritical Fluid Chromatography (Chiralpak 1A column, with 30% *i*-PrOH and 70% CO_2_ mobile phase, 40˚ C, 100 bar, 40 mL/min flow rate) to afford the title compound (1.40 g, 2.73 mmol, 48.5 %) as an off white powder. ^1^H NMR (300 MHz, DMSO-*d_6_*) δ 11.84 (s, 1H), 11.47 (s, 1H), 9.62 (t, *J*=6.4 Hz, 1H), 7.45 (s, 1H), 4.01-4.20 (m, 3H), 3.97 (d, *J*=8.85 Hz, 1H), 3.62-3.89 (m, 3H), 3.14 (t, *J*=11.8 Hz, 1H), 2.93 (d, *J*=14.3 Hz, 1H), 1.15 (d, *J*=6.2 Hz, 3H), 0.90 (d, J=6.4 Hz, 3H); ^19^F NMR (282 MHz, DMSO-*d_6_*) δ -70.1, -155.28; ^13^C NMR (101 MHz, DMSO-*d_6_*) δ 170.8, 167.6, 159.3, 152.9 (d, *J_CF_*=12.4 Hz), 151.1, 149.4, 135.3, 133.3 (d, *J_CF_*=240.0 Hz), 125.1, 124.5 (q, *J_CF_*=279.6 Hz), 115.9, 111.4, 72.1, 71.7, 64.5, 56.3 (d, *J_CF_*=9.5 Hz), 52.9, 38.3, 18.2, 18.1; UPLC RT = 0.96 min, (ES) MH^+^: 514.1 for C_21_H_20_F_4_N_5_O_6_; HRMS (ES) MH^+^ calcd for C_21_H_20_F_4_N_5_O_6_ 514.1344 found 514.1346. [α]_D_^20^ = -263 (c = 0.2; DMSO).

**5-(dimethoxymethyl)-6,7-difluorobenzo[d]isoxazole-3-carbohydrazide** A mixture of ethyl 5-(dimethoxymethyl)-6,7-difluorobenzo[d]isoxazole-3-carboxylate^15^ (10 g, 33.2 mmol) and anhydrous hydrazine (4.17 ml, 132.78 mmol) in 10 mL EtOH was stirred at RT for 3 h. Precipitated solids were filtered, rinsed with MeOH and dried in vacuo to afford the title compound (5.8 g, 60.8%). The mother liquors were combined and concentrated. The residue was dissolved in CH_2_Cl_2_, which was washed with water, dried (Na_2_SO_4_) and concentrated to give an additional 2.8 g (30.4%) of the title compound. ^1^H NMR (300 MHz, CD_2_Cl_2_) δ 8.0-8.25 (m, 2H), 5.65 (s, 1H), 4.17 (br. s., 2H), 3.38 (s, 6H); ^19^F NMR (282 MHz, DMSO-*d_6_*) δ -140.04 (d), 159.24 (d); LCMS (ES) MH^+^: C_11_H_11_F_2_N_3_O_4_.

***N*'-acetyl-5-(dimethoxymethyl)-6,7-difluorobenzo[d]isoxazole-3-carbohydrazide** HATU (7.02 g, 18.4 mmol) and diisopropylethylamine (6.45 ml, 36.9 mmol) were added sequentially to a mixture of the preceding compound (5.3 g, 18.4 mmol) and AcOH (1.11 ml, 18.4 mmol) in 30 mL DMF cooled in an ice water bath. The mixture was stirred at rt for 1.5 h. The reaction was diluted with EtOAc and washed with water. The organic layer was dried (Na_2_SO_4_) and concentrated to give the title compound (5.97 g, 98 %). ^1^H NMR (300 MHz, DMSO-*d_6_*) δ 11.00 (br. s., 1H), 10.12 (s, 1H), 7.95 (d, *J*=6.6 Hz, 1H), 5.73 (s, 1H), 3.34 (s, 6H), 1.95 (s, 3H); ^19^F NMR (282 MHz, DMSO-*d_6_*) δ -139.70 (d), 158.98 (d); LCMS (ES) MH^+^: 328 for C_13_H_13_F_2_N_3_O_5_.

**5-(dimethoxymethyl)-6,7-difluoro-3-(5-methyl-1,3,4-oxadiazol-2-yl)benzo[d]isoxazole**

A mixture of methoxycarbonylsulfamoyltriethylammonium hydroxide, inner salt (8.18 g, 34.3 mmol) and the preceding compound (5.65 g, 17.2 mmol) in 5 mL THF was stirred at rt for 36 h. Solvent was removed, and the residue was chromatographed on silica gel (30-40% EtOAc in hexanes) to afford the title compound as a solid (1.90 g, 36 %). ^1^H NMR (300 MHz, DMSO-*d_6_*) δ 8.17 (d, *J*=5.46 Hz, 1H), 5.79 (s, 1H), 3.31 (s, 6H) 2.72 (s, 3H); ^19^F NMR (282 MHz, DMSO-*d_6_*) δ -138.74 (d), 158.64 (d); LCMS (ES) MH^+^: 311.0 for C_13_H_11_F_2_N_3_O_4_.

**6,7-difluoro-3-(5-methyl-1,3,4-oxadiazol-2-yl)benzo[d]isoxazole-5-carbaldehyde** A solution of the preceding compound (2.27 g, 7.29 mmol) in 10 mL AcOH was heated at 90 °C for 7 h. Solvent was removed and the residue was dissolved in EtOAc, which was washed with water, 5% aqueous NaHCO_3_, and brine. Drying (Na_2_SO_4_) and removal of solvent afforded 1.92 g (99%) of the title compound as a solid. ^1^H NMR (300 MHz, DMSO-*d_6_*) δ 10.29 (s, 1H), 8.58 (d, *J*=5.65 Hz, 1H), 2.74 (s, 3H); ^19^F NMR (282 MHz, DMSO-*d_6_*) δ -141.32, -157.72; LCMS (ES) MH^+^: 266.0 for C_11_H_5_F_2_N_3_O_3_.

**6-((2*R*,6*R*)-2,6-dimethylmorpholino)-7-fluoro-3-(5-methyl-1,3,4-oxadiazol-2-yl)benzo[d]isoxazole-5-carbaldehyde** A mixture of 6,7-difluoro-3-(5-methyl-1,3,4-oxadiazol-2-yl)benzo[d]isoxazole-5-carbaldehyde (1.87 g, 7.04 mmol), (2*R*,6*R*)-2,6-dimethylmorpholine (0.88 mL, 7.04 mmol), and K_2_CO_3_ (1.46 g, 10.6 mmol) in butyronitrile (80 mL) and water (8 mL) was heated at reflux for 5 h. Solvent was removed. The mixture was diluted with EtOAc and washed with water and brine. The combined aqueous layers were extracted with EtOAc, which was washed with water and brine. The combined EtOAc extracts were dried (Na_2_SO_4_) and concentrated to give the title compound (2.43 g, 96 %). ^1^H NMR (300 MHz, DMSO-*d*_6_) δ 1.23 (d, *J*=6.4 Hz, 6H) 2.72 (s, 3H) 3.0-3.1 (m, 2 H) 3.39-3.5 (m, 2H) 4.10-4.25 (m, 2H) 8.37 (s, 1 H) 10.37 (s, 1H); ^19^F NMR (282 MHz, DMSO-*d_6_*) δ -144.28; LCMS (ES) MH^+^: 361.1 for C_17_H_17_FN_4_O_4_.

**(2*R*,4*S*,4a*S*)-11-Fluoro-2,4-dimethyl-8-(5-methyl-1,3,4-oxadiazol-2-yl)-2,4,4a,6-tetrahydro-1*H*,1'*H*-spiro[isoxazolo[4,5-g][1,4]oxazino[4,3-a]quinoline-5,5'-pyrimidine]-2',4',6'(3'*H*)-trione** (**8**) The preceding compound (2.4 g, 6.66 mmol) and barbituric acid (0.85 g, 6.66 mmol) in 800 mL EtOH was heated at reflux for 7 d. The mixture was concentrated, and the residue was purified by Supercritical Fluid Chromatography (Chiralpak 1A column, with 30% *i*-PrOH and 70% CO_2_ mobile phase, 40˚ C, 100 bar, 40 mL/min flow rate) to afford the title compound (2.33 g, 4.95 mmol, 74%) as an off white powder. ^1^H NMR (300 MHz, DMSO-*d*_6_) δ 11.86 (br. s., 1H), 11.49 (br. s., 1H), 7.65 (s, 1H), 4.15 (d, *J*=13.75 Hz, 1H), 4.00 (d, *J*=8.85 Hz, 1H), 3.84 (d, *J*=13.9 Hz, 2H), 3.60-3.75 (m, 1H), 3.16 (t, *J*=12.2 Hz, 1H), 2.96 (d, *J*=14.3 Hz, 1H), 2.68 (s, 3H), 1.17 (d, *J*=6.0 Hz, 3H), 0.91 (d, *J*=6.2 Hz, 3H); ^19^F NMR (282 MHz, DMSO-*d_6_*) δ -156.25; ^13^C NMR (101 MHz, DMSO-*d*_6_) δ 170.8, 167.6, 165.3, 156.2, 153.0 (d, *J_CF_*=13.2 Hz), 149.4, 144.3, 135.7, 133.3 (d, *J_CF_*=240.8 Hz), 125.5, 115.6, 110.3, 72.1, 71.7, 64.5, 56.3 (d, *J_CF_*=8.8 Hz), 52.9, 38.2, 18.2, 18.1, 10.6; UPLC RT = 0.92 min, (ES) MH^+^: 471.2 for C_21_H_20_FN_6_O_6_; HRMS (ES) MH^+^ calcd for C_21_H_20_FN_6_O_6_ 471.1423 found 471.1429. [α]_D_^20^ = -210 (c = 0.1; MeOH).

**1-[2-[(3*R*,4*S*)-4-(2,3-Dihydro-[1,4]dioxino[2,3-c]pyridin-7-ylmethylamino)-3-fluoro-1-piperidyl]ethyl]-2-oxo-quinoline-7-carbonitrile** (NBTI **3**) was prepared as described^16^.

**Determination of topoisomerase cleaved complex formation and ligation experiments.** Using a modification of published methods^17^, cleaved complexes of *S. aureus* DNA gyrase and TopoIV were formed by addition of 40 μM ciprofloxacin and AZD0914 (>20-fold excess over cleaved complex IC_50_s) with incubation for 30 min. Re-ligation was allowed for 45 min, induced by addition of 10 μl EDTA and sodium sulfate solutions, to variably buffer free Mg^2+^ concentration and balance ionic strength at approximately 0.53 M for all conditions. Reactions were quenched by adding 3 μl of 2% SDS and 1 mg/ml proteinase K (30 min at 37 °C). DNA loading dye (4 μl) was added and DNA products were quantified by gel electrophoresis. Samples (25 μl) were loaded into wells of 1% agarose gels buffered with 40 mM Tris, 20 mM acetic acid, and 1 mM EDTA at pH 8.4 and run for 18 to 20 hours at 30V, or 3 hours at 60V including 1 μg/ml ethidium bromide in gel running buffer. DNA bands from gel electrophoresis were visualized by staining with ethidium bromide (5 µg/ml in TAE) after electrophoresis. DNA was quantified from digital images acquired during UV transillumination using AlphaEase software (Genetic Technologies). Background from control wells without inhibitor was subtracted. The concentration of free Mg^2+^ for each condition in the re-ligation assays were calculated using equation (1): Starting total [Mg^2+^ ] =
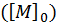
; Used [EDTA] = (
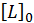
); *K*_D_ = 2.5 µM^18^

(1)
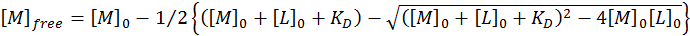


**Pharmacokinetic and distribution determinations.**

AZD0914 was analyzed in mouse, rat, dog, monkey plasma using a qualified method which was later validated for human plasma analysis. The validated human method is described below and is representative of the approach taken in other species. AZD0914 was determined in human plasma prepared with K2EDTA as an anticoagulant. AZD0914 and an internal standard (^13^C_2_, ^15^N_2_ AZD0914) were extracted from plasma with *tert*- butyl methyl ether. After evaporating to dryness under a stream of nitrogen, the residue was reconstituted and quantitated by reverse phase HPLC-MS/MS against a standard curve from 1.00 to 5000 ng/ml. Separation was achieved using a gradient which went from 55% to 85% methanol:acetonitrile (1:1) containing 0.2% formic acid as mobile phase B and 0.2% formic acid in water as mobile phase A at a flow rate of 0.60 ml/min through a Phenomonex Gemini C18 column (50 x 2.0 mm column, 5 mm particle size) heated using a Shimadzu CTO-20AC set to 40 ^o^C. AZD0914 was detected in multiple reaction monitoring (MRM) mode by positive ion electrospray ionization using a Sciex API5500 (Toronto, Canada) mass spectrometer. The ion spray voltage was set to 5500 V and turboionspray temperature was 650 ^o^C. AZD0914 was quantitated using the MRM transition 488.2 → 348.2 using a collision energy of 29 eV. The internal standard, (^13^C_2_, ^15^N_2_ AZD0914), was monitored using identical conditions except for the MRM transition of 492.2 → 348.2.

**Quantitative whole-body autoradiography.** The distribution of radioactivity in male, pigmented Lister Hood rat tissues was obtained after a single intravenous infusion over 15 minutes of ^14^C-AZD0914 at 25 mg/kg (51 μmol/kg, 5 MBq/kg) using quantitative whole-body autoradiography with phosphor-imaging technology. The potential of this compound and/or its metabolites to bind to melanin-containing tissues and/or to potentially bind covalently to tissues was also evaluated. The rats were sacrificed at 5 minutes, 1 hour, 4 hours, 24 hours, 48 hours, 7 days and 21 days after the end of infusion with enflurane (Efrane®, Abbott Laboratories, USA), and frozen in acetone, cooled to ‑70 ºC with solid CO_2_. The 7- and 21- day animals could not be quantified as no trace of radioactivity was observed. After removal of limbs and tail, the carcass was embedded, with left lateral side uppermost, in a 2.5% (w/v) aqueous solution of caramellosum natricum and frozen for at least 10 minutes. The animal blocks were stored at ‑20 ºC until sectioning. Sagittal whole-body sections (30 μm) were obtained to include organs and tissues of interest. At sectioning each block was mounted in a Leica CM3600 Cryomacrotome (Leica Microsystems GmbH, Germany) maintained at approximately ‑20 ºC. All sections were dried at ‑20 ºC for at least 1 day prior to exposure on phosphor-imaging plates. Together with two sets of calibration standards, the sections were placed on phosphor-imaging plates, which had been pre-covered with a thin plastic film. The imaging plates were exposed for 1 to 3 days at +4 to +12 ºC, enclosed in light tight cassettes in a lead shielding box to protect from environmental radiation. Following exposure, the imaging plates were scanned at a pixel size of 50 μm using FLA-7000 (FujiFilm Sverige AB, Sweden). The tissues and organs of interest were quantified using AIDA 3.5 (Raytest, Germany). The quantification limit was defined as twice the background value of the imaging plate and varied between the plates from 0.23 to 0.33 nmol/g. The various tissues and organs were identified either on the autoradiograms or on the corresponding tissue sections.

***In vitro* toxicology.** Experiments for assessing micronuclei in mouse lymphoma cells^19^ myeloid cytotoxicity^20^ and erythroid cytotoxicity^21^ were performed as described previously.

**References**

1. Basarab, G. S*. et al*. Novel DNA gyrase inhibiting spiropyrimidinetriones with a benzisoxazole scaffold – SAR and in vivo characterization. *J. Med. Chem*. **57**, 9078-9095 (2014).

2. Sahm, D., Huband, M. D. & deJonge, B. *F-264 In Vitro Activity of AZD0914, A Novel Spiropyrimidinetrione DNA Gyrase Inhibitor, Against Clinically Relevant Gram-Positive and Fastidious Gram-Negative Bacteria* (ICAAC, 2014, 2014).

3. Huband, M. D., Waites, K. D., Crabb, D. M., Kohlhoff, S. A. & Hammerschlag, M. R. *F-265 In Vitro Activity of AZD0914 and Comparators against Mycoplasma, Ureaplasma, and Chlamydia spp.* (ICAAC, 2014).

4. Jacobsson, S. *et al*. High in vitro activity of the novel spiropyrimidinetrione AZD0914, a DNA gyrase inhibitor, against multidrug-resistant *Neisseria gonorrhoeae* isolates suggests a new effective option for oral treatment of gonorrhea. *Antimicrob. Agents Chemother.* **58**, 5585-5588 (2014).

5. Huband, M. D. *et al*. In vitro antibacterial activity of AZD0914: a new spiropyrimidinetrione DNA gyrase/topoisomerase inhibitor with potent activity against Gram-positive, fasitidious Gram-negative, and atypcial bacteria. *Antimicrob. Agents and Chemother.* **59**, 467–474 (2015).

6. Su, X. *et al*. In vitro activity of the novel DNA gyrase inhibitor AZD0914 against 187 clinical *Neisseria gonorrhoeae isolates* with multi-resistance to other antimicrobials Ser. XIXth International Pathogenic Neisseria Conference (IPNC), International Pathogenic Neisseria Conference, Asheville, North Carolina, 2014.

7. Bridgland-Taylor, M. H. *et al*. Optimisation and validation of a medium-throughput electrophysiology-based hERG assay using IonWorks™ HT. *J. Pharmacol. Toxicol. Methods* **54**, 189-199 (2006).

8. Schroeder, K., Neagle, B., Trezise, D. J. & Worley, J. IonWorks™ HT: A new high-throughput electrophysiology measurement platform. *Journal of Biomolecular Screening* **8**, 50-64 (2003).

9. Balasubramanian, B. *et al*. Optimization of Ca(v)1.2 screening with an automated planar patch clamp platform. *J. Pharmacol. Toxicol. Meth.* **59**, 62-72 (2009).

10. Bridgland-Taylor, M. H. *et al*. Characterisation of an ionworks-based assay for the hKCNQ1/hKCNE1 (IKs) cardiac ion channel. *J. Pharmacol. Toxicol. Methods* **58**, 164-165 (2008).

11. Mirams, G. R. *et al*. Prediction of Thorough QT study results using action potential simulations based on ion channel screens. *J. Pharmacol. Toxicol. Methods* **70**, 246-254 (2014).

12.Knapp, S. & Patel, D. V. Bromocyclization of unsaturated thiocarbamidates. Synthesis of (±)-sporamine. *J. Am. Chem. Soc*. **105**, 6985-6986 (1983).

13. Basarab, G. S., Gowravaram, M. R., Hauck, S. I. & Zhou, F. Compounds and methods for treating bacterial infections. USP 8,889,671 (2014).

14. Korte, F. & Ludwig, G. Heterocyclics in metabolism. VIII. Synthesis of lumiflavine-4a-C14 and riboflavine-4a-C14. *Justus Liebigs Ann. Chem*. **615**, 94-99 (1958).

15. Barvian, K. *et al*. Fused, spirocyclic heteroaromatic compounds for the treatment of bacterial infections. USP 8,658,641 (2014).

16. Reck, F*. et al*. Novel N-linked aminopiperidine inhibitors of bacterial topoisomerase type II with reduced pKa: antibacterial agents with an improved safety profile. *J. Med. Chem*. **55**, 6916-6933 (2012).

17. Pierrat, O. A. & Maxwell, A. The action of the bacterial toxin microcin B17: Insight into the cleavage-religation reaction of DNA gyrase. *J. Biol. Chem*. **278**, 35016-35023 (2003).

18. Martell, A. E. & Smith, R. M. in *NIST Critical Stability Constants of Metal Complexes* (Plenum Publisher, New York, 2001).

19. Doherty, A. T., Hayes, J., Fellows, M., Kirk, S. & O’Donovan, M. A rapid, semi-automated method for scoring micronuclei in mononucleated mouse lymphoma cells. *Mutat. Res.-Gen. Tox. En.* *726,* 36-41 (2011).

20. Volpe, D. A. & Warren, M. K. Myeloid clonogenic assays for comparison of the in vitro toxicity of alkylating agents. *Toxicology in Vitro* **17***,* 271-277 (2003).

21. Boehm, D.; Bell, A. Simply red: A novel spectrophotometric erythroid proliferation assay as a tool for erythropoiesis and erythrotoxicity studies. *Biotechnology Reports* **4***,* 34-41, 2014.
